# Accelerated Stabilizer-Based Normalized Gradient Flow Algorithms for Stationary States of Bose–Einstein Condensates

**DOI:** 10.3390/e28101056

**Published:** 2026-09-25

**Authors:** Di Wang, Chongchong Song, Perry Gu, Li Yao

**Affiliations:** 1 Geely Automobile Research Institute (Ningbo) Co., Ltd., Ningbo 315336, China; di.wang14@geely.com (D.W.); songchongchong@geely.com (C.S.); 2 Institute of Aerospace Vehicle Dynamics and Control, School of Astronautics, Harbin Institute of Technology, Harbin 150001, China; 3 Zhejiang Geely Holding Group Co., Ltd., Hangzhou 310051, China; gupengyun@geely.com; 4 School of Information and Artificial Intelligence, Zhejiang University of Finance and Economics Dongfang College, Haining 314408, China

**Keywords:** Bose–Einstein condensates, Gross–Pitaevskii equations, stationary states, spectral method, normalized gradient flows, nonlocal inertia, momentum-accelerated method

## Abstract

We present an accelerated, stabilizer-based normalized gradient flow (ASNGF) framework with a nonlocal inertial (or momentum) mechanism for computing stationary states of Bose–Einstein condensates. Two schemes, ASNGF-I and ASNGF-II, are developed, and their discrete Lyapunov energy dissipation properties are established under the stated conditions. Linearized stability analyses and numerical parameter studies provide practical guidance for selecting the tunable parameters and demonstrate the important role of the nonlocal Laplacian term in the inertial dynamics. For suitable parameter scalings, the observed iteration counts are essentially insensitive to the time step over the tested range, including $\tau =10^{\pm 60}$. Numerical experiments on several Gross–Pitaevskii models show that ASNGF can substantially reduce the iteration count compared with conventional normalized gradient-flow schemes for the tested problems and parameter settings. The results also reveal that its convergence behavior is problem- and parameter-dependent. The inertial mechanism can enhance exploration of non-convex energy landscapes but does not guarantee convergence to a global minimizer. These results demonstrate the potential of ASNGF as an efficient framework for stationary-state computations in BECs.

## 1. Introduction

Bose–Einstein condensates (BECs) at temperatures *T* well below the critical condensation temperature $T_{c}$ are accurately modeled by the Gross–Pitaevskii equation (GPE), a nonlinear Schrödinger equation for the macroscopic wave function [1,2,3,4]. A fundamental computational objective is to determine stationary states of the GPE with prescribed properties, including ground states and targeted excited states. The ground state is a minimizer of the energy functional subject to the wave-function normalization constraint, whereas excited states are normalized stationary states with higher energies. Among the latter, central vortex states with integer winding numbers S>0 constitute an important class of target states [5,6]. In this work, we develop accelerated numerical algorithms for computing two-dimensional ground-state candidates and targeted excited states.

Over the past two decades, numerous numerical methods have been developed for computing stationary states of BECs. These include normalized gradient-flow methods based on the Hamiltonian of the GPE [7,8,9,10,11,12,13,14,15,16,17,18,19,20], methods for nonlinear eigenvalue problems [21,22,23,24,25,26], and constrained optimization approaches [27,28,29,30,31,32,33,34].

Normalized gradient-flow methods approach the problem from a dissipative PDE perspective. Two representative approaches are the gradient flow with discrete normalization (GFDN), also known as imaginary-time evolution [7,8,9], and the continuous normalized gradient flow (CNGF) [7,10,11]. Error analyses [35] and numerical experiments [8] have shown that certain GFDN discretizations may introduce time-step-dependent errors in the computed stationary states. The gradient flow with Lagrange multiplier (GFLM) method [18], which incorporates an explicit Lagrange multiplier, provides an alternative discretization closely related to the CNGF formulation.

Constrained optimization approaches for nonlinear eigenvalue problems include direct energy minimization by finite-element methods [28], Sobolev-gradient methods [30], regularized Newton methods [33], Riemannian optimization methods [32,34], preconditioned nonlinear conjugate-gradient (PCG) methods [31], and quasi-Newton methods such as the limited-memory BFGS (L-BFGS) method [36]. Such optimization methods can exhibit rapid local convergence once the iteration enters the basin of a stationary solution. For stationary symmetric and central vortex states, a generalized Laguerre–Hermite pseudospectral method that avoids truncation of the computational domain was developed in [37]. Rotating BEC configurations have also been studied numerically in [38].

Interacting bosonic systems such as BECs generally lead to non-convex energy landscapes containing multiple stationary and metastable states. Consequently, the stationary state reached numerically may depend on the initial data and the optimization trajectory, while locating a global minimizer can be particularly difficult when many competing configurations coexist. Purely dissipative gradient-flow methods may converge to a nearby stationary state and may explore such non-convex landscapes slowly. One approach to modifying this behavior is to introduce inertial or momentum terms into the gradient-flow dynamics:

(1) $$\begin{array}{c} \left\{\begin{array}{c} M\left(t\right){\ddot{\phi }}_{S}\left(t\right)+D\left(t\right){\dot{\phi }}_{S}\left(t\right)+\nabla E\left(\phi_{S}\left(t\right)\right)=0,\\ \phi_{S}\left(0\right)=\phi_{S}^{0},\;{\dot{\phi }}_{S}\left(0\right)={\dot{\phi }}_{S}^{0}, \end{array}\right. \end{array}$$

where M(t) and D(t) denote the inertial and damping parameters, respectively; *E* is the potential energy; and $\phi_{S}$ is the wave function. Representative momentum-based approaches include Polyak’s heavy-ball method [39,40,41] and Nesterov-type accelerated methods [42,43,44,45]. The introduction of inertia adds a kinetic contribution to the dynamical system and modifies the optimization trajectory relative to a purely overdamped gradient flow. For suitable inertial and damping parameters, the potential energy may temporarily increase while an appropriate total Lyapunov functional remains dissipative. Such underdamped dynamics can enhance exploration of the energy landscape and, in some cases, facilitate transitions across shallow energy barriers. The resulting trajectory depends on both the initial position and velocity, providing an additional degree of freedom for exploring non-convex landscapes. Related theoretical studies have also established accelerated escape from saddle-point regions for momentum-based methods under appropriate assumptions [46,47].

The gradient-flow approach is rooted in dissipative PDE theory, where the energy decreases monotonically along smooth continuous trajectories. Practical temporal discretizations, however, do not necessarily inherit an exact discrete gradient-flow structure, particularly when different terms are evaluated at different time levels. From an optimization perspective, exact preservation of the continuous gradient-flow structure is not essential for locating a stationary point of the constrained energy functional. Nevertheless, an appropriate discrete dissipation property remains valuable for understanding the stability and long-time behavior of the numerical method.

In this work, we develop an accelerated, stabilizer-based, normalized gradient flow (ASNGF) framework by introducing an underdamped dynamical system with tunable inertial, damping, and stabilization parameters. The inertial mechanism is intended to make the optimization trajectory less strongly confined to the purely overdamped descent path and thereby enhance exploration of non-convex energy landscapes. In some problems, this mechanism may facilitate escape from metastable regions and allow the iteration to reach lower-energy stationary states. Enhanced exploration, however, does not imply guaranteed global optimization. ASNGF does not provide a mathematical guarantee of escaping every higher-energy stationary state or of converging to a global minimizer. This limitation is particularly relevant for strongly non-convex problems such as rapidly rotating vortex lattices with multiple competing configurations, for which the state reached by the algorithm may depend on the initial data, symmetry-breaking perturbations, computational domain, and algorithmic parameters.

Through linearized stability analysis and finite numerical parameter studies, we provide practical guidance for selecting the tunable parameters. The resulting parameter choices should be understood as favorable choices within the ranges examined numerically rather than as globally optimal parameters. In particular, the numerical experiments reveal a marked contrast between different problem classes. For the central-vortex problem in Example 1, suitable parameter choices produce a rapid and regular energy decay toward the reference stationary energy. For the rapidly rotating multi-vortex problem considered in Section 4, the relaxation can be substantially slower and more sensitive to symmetry breaking, computational domain, and parameter choices. These observations illustrate that the convergence characteristics of ASNGF can be strongly problem- and parameter-dependent.

For the test problems and parameter settings considered in this work, ASNGF-I and ASNGF-II can require substantially fewer iterations than several conventional normalized gradient-flow schemes, including GFDN, GFLM, DSFDN, and DSFAL. At the same time, optimization methods such as PCG can remain highly competitive and may converge faster in some regimes. The objective of ASNGF is therefore not to provide a uniformly superior solver, but rather an alternative inertial normalized-gradient-flow framework that combines accelerated search behavior with a discrete dissipative structure. The numerical results further demonstrate the applicability of this framework to several GPE settings considered in this work, including central vortex states, logarithmic GPEs with rotation, and rapidly rotating multi-vortex configurations.

The remainder of this paper is organized as follows. In Section 2, we introduce two ASNGF algorithms for computing central vortex states, establish their discrete dissipation properties, and investigate practical parameter choices through linearized stability analysis and numerical experiments. We also compare their performance with GFDN, GFLM, DSFDN, DSFAL, and PCG. In Section 3, we extend the ASNGF framework to stationary states of a logarithmic GPE with angular-momentum rotation and compare the numerical results with those of existing methods. In Section 4, we apply ASNGF to rapidly rotating GP systems and examine the more challenging convergence behavior associated with multi-vortex configurations. Conclusions and directions for future work are presented in Section 5.

## 2. The Magnetic-Field-Coupled GPE and Its ASNGF Approximations

A fundamental aspect of light-ultracold gas interactions is the local-field effect (LFE)—a feedback mechanism where BECs modify light propagation to induce strong off-resonant optical coupling  [48]. This section examines the magnetic manifestation of LFE: microwave radiation coupling to internal atomic states [49]. Throughout this work, we adopt atomic units such that ℏ=m=1 and consider the focusing case, corresponding to an attractive interatomic interaction. The dynamics are governed by the magnetic-field-coupled GPE:

(2) $$\begin{array}{c} i\frac{\partial \psi }{\partial t}=-\frac{1}{2}▵\psi +g{\left|\psi \right|}^{2}\psi -H\psi ,\;x\in R^{2}, \end{array}$$

where the wave function ψ(x,t) is subject to constraint

(3) $$\begin{array}{c} N\left(\psi \left(\cdot ,t\right)\right)=\int_{R^{2}}{\left|\psi \left(x,t\right)\right|}^{2}dx=1, \end{array}$$

the attractive contact interaction *g* is a negative constant, and the magnetic field H(x) satisfies the following Poisson equation:

(4) $$\begin{array}{c} -▵H={\gamma |\psi |}^{2},\;x\in R^{2}, \end{array}$$

where γ is a microwave number. The Hamiltonian of the conservative system is given by

(5) $$\begin{array}{c} E\left(\psi \right)=\int_{R^{2}}\left[\frac{1}{2}{\left|\nabla \psi \left(x,t\right)\right|}^{2}+\frac{g}{2}{\left|\psi \left(x,t\right)\right|}^{4}-\frac{H(x,t)}{2}{\left|\psi \left(x,t\right)\right|}^{2}\right]dx. \end{array}$$

The giant self-trapped vortex annuli (VA) with large winding numbers *S* represent both fundamental quantum phenomena and valuable assets for quantum technologies [5,6,48,50,51]. This motivates one to resolve central vortex states. To compute self-trapped fundamental solitons (*S* = 0) and bright vortex annuli (*S* $\in N^{+}$), governed by (5), we adopt the following ansatz in polar coordinates (r,θ):

(6) $$\begin{array}{c} \psi \left(x,t\right)=e^{-i\mu t-iS\theta }\phi_{S}\left(r\right), \end{array}$$

where *S* ∈Z is called the winding number and $\phi_{S}\left(r\right)$ is a real-valued function of $r=\sqrt{x^{2}+y^{2}}$ in 2-D.

Given the rotational invariance of the Laplace operator and the radial symmetry of |ψ(x)|, Equation (4) implies that the magnetic field *H* is radially symmetric as well. Thus, H(x) is expressed as a function of *r* and governed by the Poisson equation,

(7) $$\begin{array}{c} -\frac{1}{r}\frac{d}{dr}(r\frac{dH}{dr})=\gamma {\left|\phi_{S}\right|}^{2},\;r\in \left[0,\infty \right). \end{array}$$

Hamiltonian (5) reduces to the following functional parameterized by winding number *S*:

(8) $$\begin{array}{c} E\left(\phi_{S}\right)=\pi \int_{0}^{\infty }\left[\left({\left|\phi_{S}^{\prime }\right|}^{2}+\frac{S^{2}}{r^{2}}{\left|\phi_{S}\right|}^{2}\right)+g{\left|\phi_{S}\right|}^{4}-H{\left|\phi_{S}\right|}^{2}\right]\;rdr, \end{array}$$

where

(9) $$\begin{array}{c} ∥\phi_{S}{∥}_{L^{2}\left(R^{+}\right)}^{2}=2\pi \int_{0}^{\infty }{\left|\phi_{S}\left(r\right)\right|}^{2}\;rdr=1. \end{array}$$

Our task here is to minimize $E(\phi_{S})$ for each fixed winding number *S*, constrained within the manifold:

(10) $$\begin{array}{c} S:=\left\{\phi_{S}\;|\;{∥\phi_{S}∥}_{L^{2}\left(R^{+}\right)}^{2}=1,E\left(\phi_{S}\right)<\infty ,\phi_{S}^{\prime }\left(0\right)=0\;\mathrm{if}\;S=0,\;\mathrm{and}\;\;\mathrm{resp}.\;\phi_{S}\left(0\right)=0\;\mathrm{if}\;S>0\right\}. \end{array}$$

Minimizing (8) subject to (9) yields the Euler–Lagrange equation:

(11) $$\begin{array}{cc} \mu \phi_{S} & =\frac{1}{2}\frac{\delta E(\phi_{S})}{\delta \phi_{S}}=-\frac{1}{2}{▵}_{r,S}\phi_{S}+g{\left|\phi_{S}\right|}^{2}\phi_{S}-H\phi_{S}, \end{array}$$

where

(12) $$\begin{array}{c} {▵}_{r,S}:=\partial_{r}^{2}+\frac{1}{r}\partial_{r}-\frac{S^{2}}{r^{2}}, \end{array}$$

and μ is the Lagrange multiplier, given by

(13) $$\begin{array}{c} \mu \left(\phi_{S}\right)=2\pi \int_{0}^{\infty }\left[\frac{1}{2}\left({\left|\phi_{S}^{\prime }\right|}^{2}+\frac{S^{2}}{r^{2}}{\left|\phi_{S}\right|}^{2}\right)+g{\left|\phi_{S}\right|}^{4}-H{\left|\phi_{S}\right|}^{2}\right]\;rdr. \end{array}$$

**Remark** **1.**  *There exists a symmetric state (S=0) and a central vortex state (S>0) of* (8) *when $g>-|g_{b}|$, where $|g_{b}|$ is listed in Table 1 [52]. When $g<-|g_{b}|$, there does not exist any symmetric or central vortex state.*

Given the rapid decay properties established by Agmon’s Theorem [53],

(14) $$\begin{array}{c} \phi_{S}\left(r\right)=o\left(e^{-\alpha r}\right)\;\mathrm{as}\;r\to \infty \;\mathrm{for}\;\alpha >0, \end{array}$$

truncating $R^{+}$ to U=[0,R] for sufficiently large R>0 is numerically justified when solving the Euler–Lagrange Equation (11). Under this truncation, Equation (7) governing the magnetic field *H* simplifies to

(15) $$\begin{array}{c} -\frac{1}{r}\frac{d}{dr}(r\frac{dH}{dr})=\gamma {\left|\phi_{S}\right|}^{2},\;r\in \left[0,R\right], \end{array}$$

subject to the Robin boundary condition at r=R given by [52,54]

(16) $$\begin{array}{c} \frac{dH}{dr}{|}_{r=R}=\frac{H(R)}{R\ln(R)}. \end{array}$$

We next introduce the accelerated, stabilizer-based normalized gradient flow (ASNGF) method with Lagrange multipliers to solve the constrained minimization problem.

### 2.1. ASNGF for Stationary States

Given time step τ>0 and $t_{n}=n\tau$ for n=0,1,⋯,∞, the GFDN method [7], a widely adopted technique for computing stationary states of BECs, takes the following form:

(17) $$\begin{array}{c} \begin{array}{cc} \; & \partial_{t}\phi_{S}=-\frac{1}{2}\frac{\delta E(\phi_{S})}{\delta \phi_{S}}=\frac{1}{2}{▵}_{r,S}\phi_{S}+\left(-g|\phi_{S}{|}^{2}+H\right)\phi_{S},\;t_{n}<t<t_{n+1},\;n\geq 0,\\ \; & \phi_{S}\left(r,t_{n+1}\right):=\phi_{S}\left(r,t_{n+1}^{+}\right)=\frac{\phi_{S}\left(r,t_{n+1}^{-}\right)}{{∥\phi_{S}\left(r,t_{n+1}^{-}\right)∥}_{L^{2}\left(R^{+}\right)}},\;n\geq 0,\\ \; & \phi_{S}\left(r,0\right)=\phi_{S}^{0}\left(r\right),\;\mathrm{with}\;{∥\phi_{S}^{0}∥}_{L^{2}\left(R^{+}\right)}^{2}=1,\;\mathrm{and}\;\phi_{S}\left(0,t\right)=0\;if\;S>0, \end{array} \end{array}$$

where $\phi_{S}\left(r,t_{n}^{\pm }\right):=\lim_{t\to t_{n}^{\pm }}\phi_{S}\left(r,t\right)$, and $\phi_{S}^{0}\left(r\right)$ is an initial guess. Essentially, it consists of a gradient descent following a gradient flow in the time interval $\left(t_{n},t_{n+1}\right]$ and then normalized to satisfy the constraint imposed on the wave function at $t_{n+1}$.

To enhance the GFDN algorithm’s computational efficiency in the relaxation dynamics, we introduce a nonlocal inertia term and modify the relaxation time in (17) by setting $M\left(t\right):=\alpha_{1}\left(t\right)-\alpha_{2}\left(t\right){▵}_{r,S}$, $D\left(t\right):=\alpha_{0}\left(t\right)$ in Equation (1), thereby resulting in the ASNGF method:

(18) $$\begin{array}{c} \begin{array}{cc} \; & \partial_{t}\phi_{S}=v\\ \; & (\alpha_{0}\left(t\right)+\left(\alpha_{1}\left(t\right)-\alpha_{2}\left(t\right){▵}_{r,S}\right)\partial_{t})v=\frac{1}{2}{▵}_{r,S}\phi_{S}+\left(-g|\phi_{S}{|}^{2}+H\right)\phi_{S}+\mu_{\phi_{S}}\left(t_{n}\right)\phi_{S}\left(r,t_{n}\right),\\ \; & \;t_{n}<t<t_{n+1},\;n\geq 0,\\ \; & \phi_{S}\left(r,t_{n+1}\right):=\phi_{S}\left(r,t_{n+1}^{+}\right)=\frac{\phi_{S}\left(r,t_{n+1}^{-}\right)}{{∥\phi_{S}\left(r,t_{n+1}^{-}\right)∥}_{L^{2}\left(R^{+}\right)}},\;n\geq 0,\\ \; & \phi_{S}\left(r,0\right)=\phi_{S}^{0}\left(r\right)\;\mathrm{with}\;{∥\phi_{S}^{0}∥}_{L^{2}\left(R^{+}\right)}^{2}=1,\;\phi_{S}\left(0,0\right)=0\;\mathrm{if}\;S>0,\\ \; & v\left(r,0\right)={\dot{\phi }}_{S}^{0}\left(r\right), \end{array} \end{array}$$

where

(19) $$\begin{array}{cc} \mu_{\phi_{S}}\left(t_{n}\right) & =\mu \left(\phi_{S}\left(\cdot ,t_{n}\right)\right) \end{array}$$

(20) $$\begin{array}{cc} \; & =2\pi \int_{0}^{\infty }\left[\frac{1}{2}\left({\left(\phi_{S}^{\prime }\left(r,t_{n}\right)\right)}^{2}+\frac{S^{2}}{r^{2}}\phi_{S}^{2}\left(r,t_{n}\right)\right)+g\phi_{S}^{4}\left(r,t_{n}\right)-H\left(r,t_{n}\right)\phi_{S}^{2}\left(r,t_{n}\right)\right]\;rdr, \end{array}$$

and the nonlocal inertia term

(21) $$\begin{array}{c} \alpha_{1}\left(t\right)-\alpha_{2}\left(t\right){▵}_{r,S},\;\mathrm{with}\;\alpha_{1}\left(t\right),\alpha_{2}\left(t\right)\geq 0, \end{array}$$

serves as a stabilizer. Standard momentum methods often produce underdamped high-frequency oscillations upon temporal discretization. The additional $-\alpha_{2}\Delta \partial_{t}\psi$ term introduces frequency-selective damping that suppresses numerical noise in high-wavenumber modes while preserving the inertial search capability on large-scale solution structures. This selective dissipation maintains numerical stability over long-time relaxation and does not compromise the algorithm’s ability to escape shallow local minima, a key advantage of momentum-based acceleration in non-convex energy landscapes. The coefficients $\alpha_{1},\alpha_{2}\geq 0$ may be fixed in practice, as demonstrated in the numerical benchmarks.

When $\alpha_{0}\left(t\right)=1$ and $\alpha_{1}\left(t\right)=\alpha_{2}\left(t\right)=0$, the ASNGF method reduces to the GFLM approach introduced in [18]. Indeed, the ASNGF method constitutes a generalization of Nesterov’s accelerated gradient method in the vanishing step size limit [55]. We believe a proper choice of time-dependent coefficients could improve computational efficiency. Some choices of the $\alpha_{i}^{\prime }s$ are discussed in this study, and a more detailed study on the time dependence of the coefficients deserves a separate study.

#### 2.1.1. Temporal Discretization of ASNGF

The temporal discretization of the ASNGF method can be formulated in several ways, particularly with respect to how the wave function normalization is imposed. In this work, we follow the temporal discretization strategy used in the GFDN/GFLM implementations [7,18] and present two ASNGF algorithms in which the wave function constraint is enforced through direct normalization. The ASNGF-I algorithm is derived by applying the backward Euler temporal discretization to the first equation and the backward-forward Euler discretization to the second equation in (18), which reads

(22) $$\begin{array}{c} \begin{array}{cc} \; & \frac{\phi_{S}^{*}-\phi_{S}^{n}}{\tau }=v^{n+1},\\ \; & \alpha_{0}v^{n+1}+\left(\alpha_{1}-\alpha_{2}{▵}_{r,S}\right)\frac{v^{n+1}-v^{n}}{\tau }=\left(\frac{1}{2}{▵}_{r,S}-\alpha \right)\phi_{S}^{*}+\left(\alpha -g|\phi_{S}^{n}{|}^{2}+H^{n}+\mu^{n}\right)\phi_{S}^{n},\\ \; & \phi_{S}^{n+1}=\frac{\phi_{S}^{*}}{∥\phi_{S}^{*}{∥}_{L^{2}}},\;n\geq 0,\;\forall S\geq 0, \end{array} \end{array}$$

where $\alpha =max\{-\frac{1}{2}(-g|u^{n}{|}^{2}+{\tilde{H}}^{n}+\mu \left(u^{n}\right)),0\}$ is the stabilization parameter introduced for numerical stability. The ASNGF-I is summarized in Algorithm 1.
**Algorithm 1:** ASNGF-I
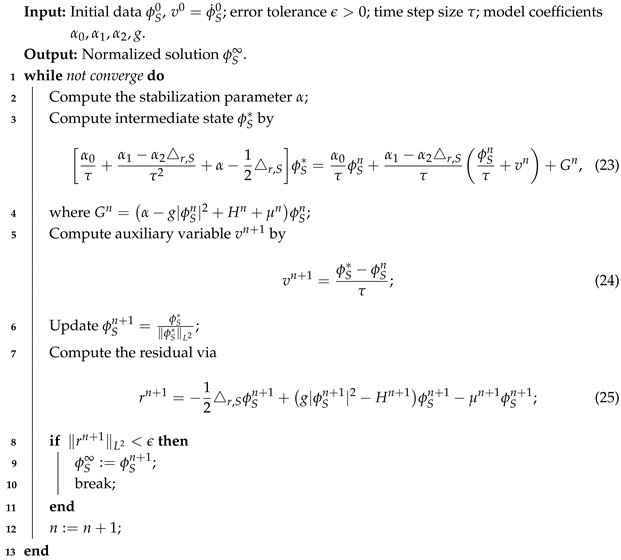


When $\alpha_{0}=1$ and $\alpha_{1}=\alpha_{2}=0$, ASNGF-I coincides with the GFLM-BF scheme of [18]. Herein, we refer to this reduced form as GFLM-I.

We define $q^{n+1}:=\tau v^{n+1}=\phi_{S}^{*}-\phi_{S}^{n}$, $q^{n}:=\tau v^{n}$, and introduce the discrete Lyapunov functional $L^{n}:=E\left(\phi_{S}^{n}\right)+\langle q^{n},\frac{\alpha_{1}-\alpha_{2}{▵}_{r,S}}{\tau^{2}}q^{n}\rangle .$ For convenience, denote $J:=\frac{\alpha_{1}-\alpha_{2}{▵}_{r,S}}{\tau^{2}}.$ We further define the radial energy norm

(26) $$\begin{array}{c} {∥u∥}_{X_{S}}^{2}:=2\pi \int_{0}^{R}\left(|u^{\prime }{\left(r\right)|}^{2}+\frac{S^{2}}{r^{2}}{\left|u\left(r\right)\right|}^{2}+{\left|u\left(r\right)\right|}^{2}\right)r\;dr, \end{array}$$

and

(27) $$\begin{array}{c} ∥\nabla_{S}{u∥}_{L^{2}}^{2}:=2\pi \int_{0}^{R}\left(|u^{\prime }{\left(r\right)|}^{2}+\frac{S^{2}}{r^{2}}{\left|u\left(r\right)\right|}^{2}\right)r\;dr. \end{array}$$

Thus, ${∥u∥}_{X_{S}}^{2}=∥\nabla_{S}{u∥}_{L^{2}}^{2}+{∥u∥}_{L^{2}}^{2}.$

The following lemma establishes a one-step dissipation property for the normalized iterates generated by the ASNGF-I scheme.

**Lemma** **1.**  Assume that the numerical trajectory $\left\{\phi_{S}^{n},\phi_{S}^{*}\right\}$ remains in a bounded subset of the radial energy space $X_{S}$ and that there exist constants $0<\rho_{0}\leq \rho_{1}<\infty$ such that $\rho_{0}\leq {∥\phi_{S}^{*}∥}_{L^{2}}\leq \rho_{1}$ for all iterations under consideration. Then, for any $\varepsilon_{1}>0$ and $\varepsilon_{2}>0$ satisfying $\varepsilon_{1}+\varepsilon_{2}<\frac{1}{2}$, there exist constants $C_{E,\varepsilon_{1}}>0$ and $C_{N,\varepsilon_{2}}>0$, independent of n, such that

(28) $$\begin{array}{cc} L^{n+1}-L^{n}\leq & -\left(\frac{1}{2}-\varepsilon_{1}-\varepsilon_{2}\right){∥\nabla_{S}q^{n+1}∥}_{L^{2}}^{2}\\ \; & -\eta_{n}{∥q^{n+1}∥}_{L^{2}}^{2}\\ \; & -\langle q^{n+1}-q^{n},J\left(q^{n+1}-q^{n}\right)\rangle , \end{array}$$

*where*

(29) $$\eta_{n}:=2\left(\frac{\alpha_{0}}{\tau }+\alpha \right)+\mu^{n}-C_{E,\varepsilon_{1}}-C_{N,\varepsilon_{2}}.$$

*Consequently, if $\eta_{n}\geq 0$, then*

(30) $$L^{n+1}\leq L^{n}.$$

*The inequality is strict whenever at least one of the dissipative terms in* (28) *is strictly positive.*

**Proof.**  From the ASNGF-I scheme and

$$q^{n+1}=\phi_{S}^{*}-\phi_{S}^{n}=\tau v^{n+1},\;q^{n}=\tau v^{n},$$

we obtain

(31) $$\begin{array}{cc} \frac{\alpha_{0}}{\tau }q^{n+1}+J\left(q^{n+1}-q^{n}\right)= & \frac{1}{2}{▵}_{r,S}\phi_{S}^{*}-\alpha q^{n+1}\\ \; & +\left(-g|\phi_{S}^{n}{|}^{2}+H^{n}\right)\phi_{S}^{n}+\mu^{n}\phi_{S}^{n}. \end{array}$$

Since *J* is self-adjoint,

(32) 2Reqn+1,J(qn+1−qn)=qn+1−qn,J(qn+1−qn)+qn+1,Jqn+1−qn,Jqn.

We first estimate the energy variation from $\phi_{S}^{n}$ to the intermediate state $\phi_{S}^{*}$. For the kinetic part,

(33) 12∥∇SϕS*∥L22−12∥∇SϕSn∥L22=Re∇Sqn+1,∇SϕS*−12∥∇Sqn+1∥L22.

Let $f\left(\phi_{S}\right)=\frac{g}{2}{∥\phi_{S}∥}_{L^{4}}^{4}.$ On bounded subsets of $X_{S}$, the nonlinear mapping $\phi_{S}\mapsto {\left|\phi_{S}\right|}^{2}\phi_{S}$ is locally Lipschitz in the corresponding Sobolev topology. Therefore, using the integral form of the Taylor remainder, there exists a constant $C_{f}>0$, independent of *n*, such that

(34) f(ϕS*)−f(ϕSn)≤2gReqn+1,|ϕSn|2ϕSn+Cf∥qn+1∥L2∥qn+1∥XS.

Similarly, let $h\left(\phi_{S}\right)=-\frac{1}{2}\langle H\left(\phi_{S}\right),{\left|\phi_{S}\right|}^{2}\rangle .$ By the elliptic regularity of the Poisson equation and the local Lipschitz continuity of the associated Poisson interaction on bounded subsets of $X_{S}$, there exists a constant $C_{h}>0$, independent of *n*, such that

(35) h(ϕS*)−h(ϕSn)≤−2Reqn+1,H(ϕSn)ϕSn+Ch∥qn+1∥L2∥qn+1∥XS.

Let $C_{E}:=C_{f}+C_{h}.$ Combining (33)–(35), we obtain

(36) E(ϕS*)−E(ϕSn)≤−2Reqn+1,12▵r,SϕS*−g|ϕSn|2−H(ϕSn)ϕSn−12∥∇Sqn+1∥L22+CE∥qn+1∥L2∥qn+1∥XS.

Since ${∥q∥}_{X_{S}}\leq \;∥\nabla_{S}{q∥}_{L^{2}}+{∥q∥}_{L^{2}},$ Young’s inequality yields, for any $\varepsilon_{1}>0$,

(37) $$\begin{array}{c} C_{E}∥q^{n+1}{∥}_{L^{2}}∥q^{n+1}{∥}_{X_{S}}\leq \varepsilon_{1}∥\nabla_{S}q^{n+1}{∥}_{L^{2}}^{2}+C_{E,\varepsilon_{1}}{∥q^{n+1}∥}_{L^{2}}^{2}, \end{array}$$

for some constant $C_{E,\varepsilon_{1}}>0$ independent of *n*.

Thus,

(38) E(ϕS*)−E(ϕSn)≤−2Reqn+1,12▵r,SϕS*−g|ϕSn|2−H(ϕSn)ϕSn−12−ε1∥∇Sqn+1∥L22+CE,ε1∥qn+1∥L22.

Using (31), (32) and (38), we obtain

(39) E(ϕS*)−E(ϕSn)+qn+1,Jqn+1−qn,Jqn≤−12−ε1∥∇Sqn+1∥L22−2α0τ+α−CE,ε1∥qn+1∥L22−qn+1−qn,J(qn+1−qn)+2Reqn+1,μnϕSn.

We now explicitly account for the normalization step. Let $s_{n+1}:={∥\phi_{S}^{*}∥}_{L^{2}}^{2}$, $\phi_{S}^{n+1}=\frac{\phi_{S}^{*}}{\sqrt{s_{n+1}}}.$ Write

(40) E(ϕ)=K(ϕ)+V(ϕ),

where

(41) $$K\left(\phi \right):=\pi \int_{0}^{R}\left(|\phi^{\prime }{\left(r\right)|}^{2}+\frac{S^{2}}{r^{2}}{\left|\phi \left(r\right)\right|}^{2}\right)r\;dr,$$

and

(42) $$V\left(\phi \right):=\pi \int_{0}^{R}\left({g|\phi \left(r\right)|}^{4}-H\left(\phi \right)\left(r\right){\left|\phi \left(r\right)\right|}^{2}\right)r\;dr.$$

Because the Poisson equation is linear in the source ${\left|\phi \right|}^{2}$, for any c>0,

(43) $$H\left(c\phi \right)=c^{2}H\left(\phi \right).$$

Consequently,

(44) $$K\left(c\phi \right)=c^{2}K\left(\phi \right),\;V\left(c\phi \right)=c^{4}V\left(\phi \right).$$

Therefore,

(45) $$E\left(\phi_{S}^{n+1}\right)=\frac{K(\phi_{S}^{*})}{s_{n+1}}+\frac{V(\phi_{S}^{*})}{s_{n+1}^{2}}.$$

Since $\phi_{S}^{*}=\phi_{S}^{n}+q^{n+1}$, $∥\phi_{S}^{n}{∥}_{L^{2}}=1,$ we have

(46) sn+1−1=2Reqn+1,ϕSn+∥qn+1∥L22.

Since $\mu^{n}$ is real,

(47) 2Reqn+1,μnϕSn=μn(sn+1−1)−μn∥qn+1∥L22.

For the normalized state $\phi_{S}^{n}$, the definition of the chemical potential yields

(48) $$\mu^{n}=K\left(\phi_{S}^{n}\right)+2V\left(\phi_{S}^{n}\right).$$

Combining (39), (45) and (47), we obtain

(49) $$\begin{array}{cc} L^{n+1}-L^{n}\leq & -\left(\frac{1}{2}-\varepsilon_{1}\right){∥\nabla_{S}q^{n+1}∥}_{L^{2}}^{2}\\ \; & -\left[2\left(\frac{\alpha_{0}}{\tau }+\alpha \right)+\mu^{n}-C_{E,\varepsilon_{1}}\right]{∥q^{n+1}∥}_{L^{2}}^{2}\\ \; & -\langle q^{n+1}-q^{n},J\left(q^{n+1}-q^{n}\right)\rangle +R_{N}^{n+1}, \end{array}$$

where

(50) $$\begin{array}{cc} R_{N}^{n+1}:= & \mu^{n}\left(s_{n+1}-1\right)+\left(\frac{1}{s_{n+1}}-1\right)K\left(\phi_{S}^{*}\right)\\ \; & +\left(\frac{1}{s_{n+1}^{2}}-1\right)V\left(\phi_{S}^{*}\right). \end{array}$$

Using

$$\frac{1}{s}-1=-\frac{s-1}{s},\;\frac{1}{s^{2}}-1=-\frac{\left(s-1)(s+1\right)}{s^{2}},$$

we obtain the exact factorization

(51) $$\begin{array}{c} R_{N}^{n+1}=\left(s_{n+1}-1\right)\left[\mu^{n}-\frac{K(\phi_{S}^{*})}{s_{n+1}}-\frac{s_{n+1}+1}{s_{n+1}^{2}}V\left(\phi_{S}^{*}\right)\right]. \end{array}$$

Using (48), the second factor can be decomposed as

(52) $$\begin{array}{cc} \; & \mu^{n}-\frac{K(\phi_{S}^{*})}{s_{n+1}}-\frac{s_{n+1}+1}{s_{n+1}^{2}}V\left(\phi_{S}^{*}\right)\\ = & K\left(\phi_{S}^{n}\right)-K\left(\phi_{S}^{*}\right)+2\left[V\left(\phi_{S}^{n}\right)-V\left(\phi_{S}^{*}\right)\right]\\ \; & +\left(1-\frac{1}{s_{n+1}}\right)K\left(\phi_{S}^{*}\right)\\ \; & +\left(2-\frac{s_{n+1}+1}{s_{n+1}^{2}}\right)V\left(\phi_{S}^{*}\right). \end{array}$$

Since $s_{n+1}\geq \rho_{0}^{2}>0,$ we have

(53) $$\left|1-\frac{1}{s_{n+1}}\right|\leq \frac{1}{\rho_{0}^{2}}\left|s_{n+1}-1\right|.$$

Moreover,

(54) $$2-\frac{s_{n+1}+1}{s_{n+1}^{2}}=\frac{\left(s_{n+1}-1\right)\left(2s_{n+1}+1\right)}{s_{n+1}^{2}}.$$

Since $s_{n+1}$ is uniformly bounded above and below, there exists $C_{s}>0$, independent of *n*, such that

(55) $$\left|2-\frac{s_{n+1}+1}{s_{n+1}^{2}}\right|\leq C_{s}\left|s_{n+1}-1\right|.$$

From (46),

(56) $$|s_{n+1}-1|\leq 2∥q^{n+1}{∥}_{L^{2}}+{∥q^{n+1}∥}_{L^{2}}^{2}.$$

Since the numerical trajectory is bounded in $X_{S}$, there exists $C_{0}>0$, independent of *n*, such that

(57) $$|s_{n+1}-1|\leq C_{0}{∥q^{n+1}∥}_{L^{2}}.$$

On bounded subsets of $X_{S}$, the functionals *K* and *V* are locally Lipschitz. Hence,

(58) $$|K\left(\phi_{S}^{n}\right)-K\left(\phi_{S}^{*}\right)|\;+\;|V\left(\phi_{S}^{n}\right)-V\left(\phi_{S}^{*}\right)|\;\leq C∥q^{n+1}{∥}_{X_{S}}.$$

Moreover, $K(\phi_{S}^{*})$ and $V(\phi_{S}^{*})$ are uniformly bounded. Combining these estimates with (57), we obtain

(59) $$\begin{array}{c} \left|\mu^{n}-\frac{K(\phi_{S}^{*})}{s_{n+1}}-\frac{s_{n+1}+1}{s_{n+1}^{2}}V\left(\phi_{S}^{*}\right)\right|\leq C_{1}{∥q^{n+1}∥}_{X_{S}}, \end{array}$$

where $C_{1}>0$ is independent of *n*.

Consequently,

(60) $$|R_{N}^{n+1}|\;\leq C∥q^{n+1}{∥}_{L^{2}}{∥q^{n+1}∥}_{X_{S}}.$$

Using Young’s inequality once more, for every $\varepsilon_{2}>0$, there exists $C_{N,\varepsilon_{2}}>0$ such that

(61) $$\begin{array}{cc} |R_{N}^{n+1}|\;\leq & \varepsilon_{2}∥\nabla_{S}q^{n+1}{∥}_{L^{2}}^{2}+C_{N,\varepsilon_{2}}{∥q^{n+1}∥}_{L^{2}}^{2}. \end{array}$$

Substituting (61) into (49) yields

(62) $$\begin{array}{cc} L^{n+1}-L^{n}\leq & -\left(\frac{1}{2}-\varepsilon_{1}-\varepsilon_{2}\right){∥\nabla_{S}q^{n+1}∥}_{L^{2}}^{2}\\ \; & -\left[2\left(\frac{\alpha_{0}}{\tau }+\alpha \right)+\mu^{n}-C_{E,\varepsilon_{1}}-C_{N,\varepsilon_{2}}\right]{∥q^{n+1}∥}_{L^{2}}^{2}\\ \; & -\langle q^{n+1}-q^{n},J\left(q^{n+1}-q^{n}\right)\rangle . \end{array}$$

Therefore, if $\eta_{n}=2\left(\frac{\alpha_{0}}{\tau }+\alpha \right)+\mu^{n}-C_{E,\varepsilon_{1}}-C_{N,\varepsilon_{2}}\geq 0$ and $\varepsilon_{1}+\varepsilon_{2}<\frac{1}{2},$ then

(63) $$L^{n+1}\leq L^{n}.$$

This completes the proof.    □

To understand the influence of the parameters: $\alpha ,\alpha_{0},\alpha_{1},\alpha_{2}$ on the algorithm, we perform a linear stability analysis for Algorithm 1 with $\phi_{S}^{*}=\phi_{S}^{n+1}$. Consider small perturbations around a steady state. Neglecting nonlinear terms and the projection operator for the normalization, we arrive at the linearized iteration scheme as follows:

(64) $$\begin{array}{c} \begin{array}{cc} \frac{\phi_{S}^{*}-\phi_{S}^{n}}{\tau } & =v^{n+1},\\ \alpha_{0}v^{n+1}+\left(\alpha_{1}-\alpha_{2}{▵}_{r,S}\right)\frac{v^{n+1}-v^{n}}{\tau } & =\left(\frac{1}{2}{▵}_{r,S}-\alpha \right)\phi_{S}^{n+1}+ℵ\phi_{S}^{n}, \end{array} \end{array}$$

where *ℵ* is a coefficient incorporating the linearized chemical potential. To analyze the stability and convergence, we consider the linearized equations obtained by freezing the coefficient *ℵ*.

We introduce a plane wave ansatz

(65) $$\begin{array}{c} \phi_{S,k}^{n}=\lambda^{n}e^{-ik\cdot x}, \end{array}$$

where k=|k| is the wavenumber and λ is the amplification factor. Substituting (65) into (64), we obtain the characteristic equation of λ:

(66) $$\xi \lambda^{2}-\vartheta \lambda +\sigma =0,$$

where

(67) $$\begin{array}{cc} \sigma & =\alpha_{1}+\alpha_{2}k^{2}, \end{array}$$

(68) $$\begin{array}{cc} \xi & =\sigma +\alpha_{0}\tau +\tau^{2}\left(\frac{k^{2}}{2}+\alpha \right), \end{array}$$

(69) $$\begin{array}{cc} \vartheta & =2\sigma +\alpha_{0}\tau +\tau^{2}\left(\alpha +ℵ\right). \end{array}$$

Applying the Schur–Cohn criterion to (66), all roots satisfy |λ|<1 if and only if

(70) $$\begin{array}{c} |\sigma |<\xi ,\;\mathrm{and}\;\;|\vartheta |<\sigma +\xi . \end{array}$$

Since σ>0 and ξ>σ, the first condition is trivially satisfied. The second condition leads to two inequalities:

- If ϑ≥0, then ϑ<σ+ξ gives $ℵ<\frac{k^{2}}{2}$.
- If ϑ<0, then −ϑ<σ+ξ gives $ℵ>-\left(\frac{4\sigma }{\tau^{2}}+\frac{2\alpha_{0}}{\tau }+\frac{k^{2}}{2}+2\alpha \right)$.

The most restrictive case for the first inequality is k=0, which requires ℵ<0. For k>0, $\frac{k^{2}}{2}>0$, so ℵ<0 automatically ensures $ℵ<\frac{k^{2}}{2}$. The second inequality gives a lower bound that is very negative for small τ. For the nonlinear gradient flow, however, there is no guarantee that *ℵ* must be negative. For the purpose of developing a selection strategy for the $\alpha^{\prime }s$ in the algorithm, we focus on the regime in which ℵ<0.

We now discuss the spectral radius and its dependence on the parameters when ℵ<0. The convergence rate of the linear scheme is determined by the spectral radius $\max_{k}\left|\lambda \left(k\right)\right|$. A smaller spectral radius implies faster convergence. To understand how it depends on the parameters, we examine the discriminant of (66):

(71) $$\begin{array}{cc} \Delta (k) & =\vartheta^{2}-4\sigma \xi \\ \; & =\tau^{2}\left\{{\left[\alpha_{0}+\tau \left(\alpha +ℵ\right)\right]}^{2}+4\alpha_{1}ℵ+2\left(2\alpha_{2}ℵ-\alpha_{1}\right)k^{2}-2\alpha_{2}k^{4}\right\}. \end{array}$$

The roots (real or complex) and their magnitudes vary with *k*.

Low-frequency limit (k→0).

For small *k*, if Δ(0)≤0, the roots are complex conjugates with magnitude

(72) $$\begin{array}{c} \left|\lambda \right|=\sqrt{\frac{\sigma }{\xi }}\approx \sqrt{\frac{\alpha_{1}}{\alpha_{1}+\alpha_{0}\tau +\alpha \tau^{2}}}. \end{array}$$

This value decreases as $\alpha_{0}$ or α increase. When Δ(0)>0, the roots are real and the larger one may be close to 1, leading to slow decay. To achieve fast decay, we aim to make Δ(0)≤0 by choosing $\alpha_{0}$ and α appropriately.

High-frequency limit (k→∞).

For large *k*, assuming $\alpha_{2}>0$, the discriminant is dominated by $-2\alpha_{2}k^{4}\tau^{2}$, so it is negative. Then the roots are complex with magnitude

(73) $$\begin{array}{c} \left|\lambda \right|=\sqrt{\frac{\sigma }{\xi }}\approx \sqrt{\frac{\alpha_{2}k^{2}}{\alpha_{2}k^{2}+\frac{\tau^{2}}{2}k^{2}}}=\sqrt{\frac{\alpha_{2}}{\alpha_{2}+\frac{\tau^{2}}{2}}}. \end{array}$$

Thus, the high-frequency modes have a magnitude that approaches a constant less than 1, but this constant tends to 1 as $\alpha_{2}$ increases. Hence, a small $\alpha_{2}$ is desirable for rapid damping of high-frequency oscillations.

To minimize the spectral radius, we must balance the decay rates across all wavenumbers. A common strategy is to first tune $\alpha_{0}$ and α so that the low-frequency modes are critically damped, i.e., Δ(0)=0. From (71), setting Δ(0)=0 gives

(74) $$\alpha_{0}+\tau \left(\alpha +ℵ\right)=\pm 2\sqrt{-\alpha_{1}ℵ}.$$

Choosing the positive branch (which typically yields a larger $\alpha_{0}$) leads to

(75) $$\alpha_{0}+\alpha \tau =2\sqrt{-\alpha_{1}ℵ}-ℵ\tau .$$

Under this condition, the low-frequency amplification factor becomes

(76) $${\left|\lambda \right|}_{\mathrm{low}}=\frac{\sqrt{\alpha_{1}}}{\sqrt{\alpha_{1}}+\sqrt{-ℵ}\tau }.$$

This value decreases as $\alpha_{1}$ decreases, but $\alpha_{1}$ also appears in the high-frequency behavior.

For high frequencies, the magnitude is given by

(77) $${\left|\lambda \right|}_{\mathrm{high}}=\sqrt{\frac{\alpha_{2}}{\alpha_{2}+\tau^{2}/2}}.$$

To ensure that the high-frequency modes do not become the bottleneck, we require ${\left|\lambda \right|}_{\mathrm{high}}\leq {\left|\lambda \right|}_{\mathrm{low}}$. This yields an upper bound on $\alpha_{2}$:

(78) $$\sqrt{\frac{\alpha_{2}}{\alpha_{2}+\tau^{2}/2}}\leq \frac{\sqrt{\alpha_{1}}}{\sqrt{\alpha_{1}}+\sqrt{-ℵ}\tau }.$$

Solving for $\alpha_{2}$ gives

(79) $$\alpha_{2}\leq \frac{\tau^{2}}{2}\cdot \frac{\alpha_{1}}{{\left(\sqrt{\alpha_{1}}+\sqrt{-ℵ}\tau \right)}^{2}-\alpha_{1}}.$$

To minimize the overall spectral radius, we would like to make both ${\left|\lambda \right|}_{\mathrm{low}}$ and ${\left|\lambda \right|}_{\mathrm{high}}$ as small as possible.

Note that $\alpha_{2}$ can be zero, in which case high-frequency modes are perfectly damped (the magnitude tends to zero as k→∞). But taking $\alpha_{2}=0$ removes the inertial term with the Laplacian, which may affect the overall convergence. In practice, a common simplification is to set $\alpha_{1}=1$, $\alpha_{2}=0$ and then choose $\alpha ,\alpha_{0}$ such that the stability condition for the highest resolved wavenumber $k_{\max}$ is satisfied. For $\alpha_{2}=0$, the high-frequency modes are damped very quickly (their magnitude tends to 0 as *k* increases), so the spectral radius is determined by the low-frequency modes. The stability condition from the von Neumann analysis for $k_{\max}$ becomes

(80) $$\frac{\tau^{2}k_{\max}^{2}}{2}\leq 1+\alpha_{0}\tau ,$$

which imposes a relation between τ, $k_{\max}$, and $\alpha_{0}$. For a fixed τ, increasing $\alpha_{0}$ allows larger $k_{\max}$ but also slows down low-frequency decay according to (76) (with α=0 and $\alpha_{1}=1$). Hence, a trade-off exists; the “optimal” $\alpha_{0}$ can be found by minimizing $\max\{|\lambda {|}_{\mathrm{low}},|\lambda {|}_{\mathrm{high}}\}$ over the discrete set of wavenumbers. This is a minimax problem that can be solved numerically for a given τ.

The linearized stability analysis suggests that the smaller $\alpha_{1}$ and $\alpha_{2}$ favor a smaller growth rate. However, (75) imposes a constraint among $\alpha_{0},\alpha$ and $\alpha_{1}$, and (79) imposes one between $\alpha_{1}$ and $\alpha_{2}$. If we choose $\alpha_{0}=O\left(\tau \right),\alpha_{1}=O\left(\tau^{2}\right),\alpha_{2}=O\left(\tau^{2}\right),\mathrm{and}\;{\dot{\phi }}_{S}^{0}=0$, both constraints could be satisfied. Moreover, the number of iterations required for the ASNGF-I algorithm to converge to the steady state becomes independent of τ with this choice. This serves as the rationale for the choice of the parameter values in our numerical experiments. With this choice of the parameters, τ can take on extreme values like $10^{\pm 60}$. It can be readily proven that the convergent state of the ASNGF-I scheme corresponds precisely to the eigenfunction of the Euler–Lagrange equation.

Employing the backward-Euler discretization on the first equation and the forward-Euler discretization on the second equation in (18) produces the ASNGF-II algorithm (Algorithm 2), which reads

(81) $$\begin{array}{c} \begin{array}{cc} \; & \frac{\phi_{S}^{*}-\phi_{S}^{n}}{\tau }=v^{n+1},\\ \; & \alpha_{0}v^{n+1}+\left(\alpha_{1}-\alpha_{2}{▵}_{r,S}\right)\frac{v^{n+1}-v^{n}}{\tau }=\frac{1}{2}{▵}_{r,S}\phi_{S}^{n}+\left(-g|\phi_{S}^{n}{|}^{2}+H^{n}+\mu^{n}\right)\phi_{S}^{n},\\ \; & \phi_{S}^{n+1}=\frac{\phi_{S}^{*}}{∥\phi_{S}^{*}{∥}_{L^{2}}},\;n\geq 0,\;\forall S\geq 0. \end{array} \end{array}$$

Setting $\alpha_{0}=1$ and $\alpha_{1}=\alpha_{2}=0$ reduces ASNGF-II to the GFLM-FE formulation in [18]. This particular scheme is called GFLM-II below.

The following lemma establishes a one-step dissipation property for the normalized iterates generated by the spatially discretized ASNGF-II scheme.
**Algorithm 2:** ASNGF-II
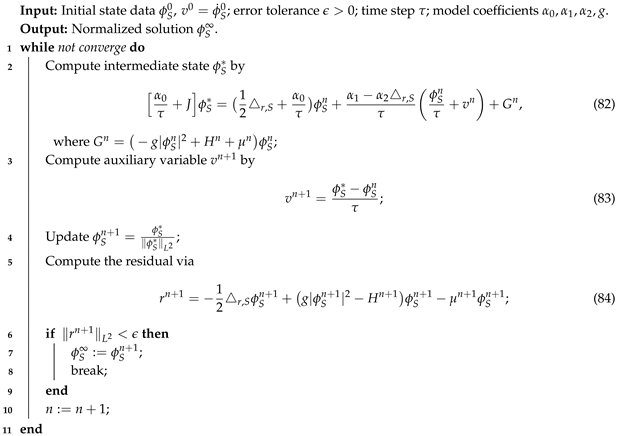

**Lemma** **2.**  Let $X_{S,h}\subset X_{S}$ be a fixed finite-dimensional spatial approximation space. Assume that the numerical trajectory $\left\{\phi_{S}^{n},\phi_{S}^{*}\right\}\subset X_{S,h}$ remains in a bounded subset of $X_{S}$ and that there exist constants $0<\rho_{0}\leq \rho_{1}<\infty$ such that $\rho_{0}\leq {∥\phi_{S}^{*}∥}_{L^{2}}\leq \rho_{1}$ for all iterations under consideration. In addition, assume that the inverse estimate

(85) $$∥w_{h}{∥}_{X_{S}}\leq C_{\mathrm{inv}}\left(h\right){∥w_{h}∥}_{L^{2}},\;w_{h}\in X_{S,h},$$

*holds, where $C_{\mathrm{inv}}\left(h\right)>0$ is independent of the iteration index n. Then there exist constants $C_{E}\left(h\right)>0$ and $C_{N}\left(h\right)>0$, independent of n, such that*

(86) $$\begin{array}{c} L^{n+1}-L^{n}\leq -\eta_{n}{∥q^{n+1}∥}_{L^{2}}^{2}-\langle q^{n+1}-q^{n},J\left(q^{n+1}-q^{n}\right)\rangle , \end{array}$$

*where*

(87) $$\eta_{n}:=\frac{2\alpha_{0}}{\tau }+\mu^{n}-C_{E}\left(h\right)-C_{N}\left(h\right).$$

*Consequently, if $\eta_{n}\geq 0$, then*

(88) $$L^{n+1}\leq L^{n}.$$

*Moreover, the inequality is strict whenever at least one of the dissipative terms in* (86) *is strictly positive.*

**Proof.**  From the ASNGF-II scheme and $q^{n+1}=\phi_{S}^{*}-\phi_{S}^{n}=\tau v^{n+1}$, $q^{n}=\tau v^{n},$ we obtain

(89) $$\begin{array}{c} \frac{\alpha_{0}}{\tau }q^{n+1}+J\left(q^{n+1}-q^{n}\right)=\frac{1}{2}{▵}_{r,S}\phi_{S}^{n}+\left(-g|\phi_{S}^{n}{|}^{2}+H\left(\phi_{S}^{n}\right)\right)\phi_{S}^{n}+\mu^{n}\phi_{S}^{n}. \end{array}$$

Since *J* is self-adjoint,

(90) 2Reqn+1,J(qn+1−qn)=qn+1−qn,J(qn+1−qn)+qn+1,Jqn+1−qn,Jqn.

We first estimate the energy variation from $\phi_{S}^{n}$ to the intermediate state $\phi_{S}^{*}$. The first variation of the energy satisfies

(91) DE(ϕSn)[qn+1]=−2Reqn+1,12▵r,S−g|ϕSn|2+H(ϕSn)ϕSn.

On the bounded set containing the numerical trajectory, the second variation of *E* is uniformly bounded as a bilinear form on $X_{S}\times X_{S}$. Hence, there exists a constant $M_{E}\left(h\right)>0$, independent of *n*, such that

(92) $$\left|D^{2}E\left(\phi \right)\left[w,w\right]\right|\leq M_{E}\left(h\right){∥w∥}_{X_{S}}^{2}$$

for all relevant *ϕ* and $w\in X_{S,h}$.

Using the integral form of Taylor’s theorem,

(93) $$\begin{array}{c} E\left(\phi_{S}^{*}\right)-E\left(\phi_{S}^{n}\right)=DE\left(\phi_{S}^{n}\right)\left[q^{n+1}\right]+\int_{0}^{1}\left(1-\theta \right)D^{2}E\left(\phi_{S}^{n}+\theta q^{n+1}\right)\left[q^{n+1},q^{n+1}\right]\;d\theta , \end{array}$$

and therefore

(94) $$\begin{array}{cc} E\left(\phi_{S}^{*}\right)-E\left(\phi_{S}^{n}\right)\leq & DE\left(\phi_{S}^{n}\right)\left[q^{n+1}\right]+\frac{M_{E}\left(h\right)}{2}{∥q^{n+1}∥}_{X_{S}}^{2}. \end{array}$$

Using the inverse estimate (85), we have

(95) E(ϕS*)−E(ϕSn)≤−2Reqn+1,12▵r,S−g|ϕSn|2+H(ϕSn)ϕSn+CE(h)∥qn+1∥L22,

where $C_{E}\left(h\right):=\frac{M_{E}\left(h\right)}{2}C_{\mathrm{inv}}{\left(h\right)}^{2}.$

Taking the real part of the $L^{2}$ inner product of (89) with $2q^{n+1}$, and using (90) and (95), we obtain

(96) E(ϕS*)−E(ϕSn)+qn+1,Jqn+1−qn,Jqn≤−2α0τ−CE(h)∥qn+1∥L22−qn+1−qn,J(qn+1−qn)+2Reqn+1,μnϕSn.

The normalization step is handled exactly as in the proof of Lemma 1. More precisely, setting $s_{n+1}={∥\phi_{S}^{*}∥}_{L^{2}}^{2}$, $\phi_{S}^{n+1}=\frac{\phi_{S}^{*}}{\sqrt{s_{n+1}}},$ and using $\mu^{n}=K\left(\phi_{S}^{n}\right)+2V\left(\phi_{S}^{n}\right)$, the multiplier term and the energy change induced by normalization can be combined into the same normalization remainder $R_{N}^{n+1}$ as in Lemma 1. Therefore,

(97) $$\begin{array}{c} L^{n+1}-L^{n}\leq -\left(\frac{2\alpha_{0}}{\tau }+\mu^{n}-C_{E}\left(h\right)\right){∥q^{n+1}∥}_{L^{2}}^{2}-\langle q^{n+1}-q^{n},J\left(q^{n+1}-q^{n}\right)\rangle +R_{N}^{n+1}. \end{array}$$

As proved in Lemma 1, the boundedness assumptions and $\rho_{0}\leq {∥\phi_{S}^{*}∥}_{L^{2}}\leq \rho_{1}$ imply

$$|R_{N}^{n+1}|\;\leq C∥q^{n+1}{∥}_{L^{2}}{∥q^{n+1}∥}_{X_{S}}.$$

Using the inverse estimate once again gives

(98) $$|R_{N}^{n+1}|\;\leq C_{N}\left(h\right){∥q^{n+1}∥}_{L^{2}}^{2},$$

where $C_{N}\left(h\right)>0$ is independent of *n*.

Substituting (98) into (97), we obtain

(99) $$\begin{array}{c} L^{n+1}-L^{n}\leq -\left(\frac{2\alpha_{0}}{\tau }+\mu^{n}-C_{E}\left(h\right)-C_{N}\left(h\right)\right){∥q^{n+1}∥}_{L^{2}}^{2}-\langle q^{n+1}-q^{n},J\left(q^{n+1}-q^{n}\right)\rangle . \end{array}$$

Hence, if $\eta_{n}=\frac{2\alpha_{0}}{\tau }+\mu^{n}-C_{E}\left(h\right)-C_{N}\left(h\right)\geq 0$, then

$$L^{n+1}\leq L^{n}.$$

This completes the proof.    □

**Remark** **2.**  *The condition*

$$\eta_{n}=\frac{2\alpha_{0}}{\tau }+\mu^{n}-C_{E}\left(h\right)-C_{N}\left(h\right)\geq 0$$

*in Lemma 2 is a sufficient condition for the one-step dissipation of the discrete Lyapunov functional, rather than a necessary condition for the convergence of the ASNGF-II scheme.*

*In particular, the parameter choice $\alpha_{0}=0$, adopted in the numerical experiments below, is not claimed to satisfy the sufficient condition of Lemma 2 for every iteration. Instead, this choice is selected empirically according to its observed numerical performance. Therefore, the theoretical stability condition in Lemma 2 and the numerical parameter configuration used in the experiments should be understood separately.*

Analogously, a linear stability analysis of Algorithm 2 is performed by inserting the plane-wave ansatz (65) into the linearized Algorithm 2, yielding a characteristic polynomial for the amplification factor:

(100) $$\left(\sigma +\alpha_{0}\tau \right)\lambda^{2}-\left(2\sigma +\alpha_{0}\tau +\xi \tau^{2}\right)\lambda +\sigma =0,$$

where $\sigma =\alpha_{1}+\alpha_{2}k^{2},\;\xi =-\frac{k^{2}}{2}+ℵ$.

The convergence rate is determined by the spectral radius $\max_{k}\left|\lambda \left(k\right)\right|$. The two roots $\lambda_{1,2}$ of (100) satisfy

(101) $$\begin{array}{c} \lambda_{1}\lambda_{2}=\frac{\sigma }{\sigma +\alpha_{0}\tau }<1,\;\lambda_{1}+\lambda_{2}=\frac{2\sigma +\alpha_{0}\tau +\xi \tau^{2}}{\sigma +\alpha_{0}\tau } \end{array}$$

The magnitude of the roots depends on the discriminant

(102) $$\begin{array}{c} \Delta ={\left(2\sigma +\alpha_{0}\tau +\xi \tau^{2}\right)}^{2}-4\sigma \left(\sigma +\alpha_{0}\tau \right). \end{array}$$

We once again focus on the case ℵ<0.

Low-frequency limit (k→0).

Here $\sigma \approx \alpha_{1}$ and ξ≈ℵ. Equation (100) simplifies to

(103) $$\begin{array}{c} \left(\alpha_{1}+\alpha_{0}\tau \right)\lambda^{2}-\left(2\alpha_{1}+\alpha_{0}\tau +ℵ\tau^{2}\right)\lambda +\alpha_{1}=0. \end{array}$$

The roots are determined by $\alpha_{0}$ and $\alpha_{1}$. So long as $\alpha_{1}$ is sufficiently large, the roots are complex with a magnitude less than 1.

High-frequency limit (k→∞).

In this case, $\sigma \approx \alpha_{2}k^{2}$ and $\xi \approx -\frac{k^{2}}{2}$. Equation (100) becomes

(104) $$\begin{array}{c} \left(\alpha_{2}k^{2}+\alpha_{0}\tau \right)\lambda^{2}-\left(2\alpha_{2}k^{2}+\alpha_{0}\tau -\frac{k^{2}}{2}\tau^{2}\right)\lambda +\alpha_{2}k^{2}=0. \end{array}$$

Dividing by $k^{2}$ and taking k→∞ gives

(105) $$\begin{array}{c} \;\alpha_{2}\lambda^{2}-\left(2\alpha_{2}-\frac{\tau^{2}}{2}\right)\lambda +\alpha_{2}=0. \end{array}$$

Its discriminant is

(106) $$\begin{array}{c} \Delta =\tau^{2}\left(\tau^{2}/4-2\alpha_{2}\right). \end{array}$$

If $\alpha_{2}\geq \tau^{2}/8$, the high-frequency modes have modulus 1 and do not decay, so the convergence rate is limited by low-frequency modes.

In practice, the grid restricts the maximum wave number $k_{max}$, so all discrete wave numbers must be considered. The convergence rate is governed by the slowest decaying mode, i.e., the largest |λ| over *k*. By adjusting $\alpha_{0},\alpha_{1},\alpha_{2}$, we can minimize this maximum, which typically involves balancing low- and high-frequency decay.

The linear analysis allows us to develop a strategy to choose proper $\alpha_{0},\alpha_{1},\alpha_{2}$ in practical implementation. If we choose $\alpha_{0}=O\left(\tau \right)$, and $\alpha_{1}=\alpha_{2}=O\left(\tau^{2}\right)$ while assuming ${\dot{\phi }}_{S}^{0}=0$, we could have a small growth rate λ, thereby improving the overall convergence rate of the linearized algorithm. Compared to other gradient flow methods, the above two new schemes require only minimal additional vector additions without increasing the complexity of matrix-vector multiplication operations.

#### 2.1.2. Spatial Discretization via the Legendre–Galerkin Spectral Method for Central Vortex States

Central vortex states typically exhibit fine-scale spatial structures, necessitating a high-resolution spatial discretization of the governing GPE system. In this study, we employ the Legendre–Galerkin spectral method [56] to conduct the spatial discretization. We map [0,R] into [−1,1] using transformation $r=\frac{R}{2}\left(x+1\right)$, where x∈I=[−1,1] and denote $u\left(x\right)=\phi_{S}\left(\frac{R}{2}\left(x+1\right)\right)$, $v\left(x\right)={\dot{\phi }}_{S}\left(\frac{R}{2}\left(x+1\right)\right)$, and $\tilde{H}\left(x\right)=H\left(r\right)$. Viewing *u* and *v* as functions of (x,t) for x∈[−1,1],t>0, we reformulate (18) as follows:

(107) $$\begin{array}{c} \begin{array}{cc} \; & \partial_{t}u=v,\;t_{n}<t<t_{n+1},\;n\geq 0,\\ \; & \left(\alpha_{0}+\left(\alpha_{1}-\frac{4\alpha_{2}}{R^{2}}{▵}_{x,S}\right)\partial_{t}\right)v=\frac{2}{R^{2}}{▵}_{x,S}u-\left(gu^{2}-\tilde{H}\right)u+\mu \left(u(t_{n})\right)u\left(t_{n}\right),\\ & \;t_{n}<t<t_{n+1},\;n\geq 0,\\ \; & u\left(x,t_{n+1}\right):=\frac{u(x,t_{n+1}^{+})}{∥u\left(x,t_{n+1}^{+}\right){∥}_{L^{2}\left(I\right)}},\;n\geq 0,\\ \; & u^{0}\left(x\right)=\phi_{S}^{0}\left(r\right),\;v^{0}\left(x\right)={\dot{\phi }}_{S}^{0}\left(r\right), \end{array} \end{array}$$

where ${∥f∥}_{L^{2}\left(I\right)}^{2}=\frac{\pi R^{2}}{2}\int_{-1}^{1}f^{2}\left(x\right)\left(x+1\right)dx$, and

(108) $$\begin{array}{c} {▵}_{x,S}:=\partial_{x}^{2}+\frac{1}{x+1}\partial_{x}-\frac{S^{2}}{{\left(x+1\right)}^{2}}. \end{array}$$

The chemical potential μ at time $t_{n}$ is reformulated as follows:

(109) $$\begin{array}{cc} \mu (u\left(t_{n}\right))= & \pi \int_{-1}^{1}\left[{\left(\partial_{x}u\left(t_{n}\right)\right)}^{2}+\frac{S^{2}}{{\left(x+1\right)}^{2}}u^{2}\left(t_{n}\right)+\frac{R^{2}}{2}\left(gu^{2}\left(t_{n}\right)-\tilde{H}\left(t_{n}\right)\right)u^{2}\left(t_{n}\right)\right]\;\left(x+1\right)dx. \end{array}$$

Given an integer *N*, let $P_{N}$ denote the space of polynomials of degree less than or equal to *N*, and define two function subspaces

(110) $$\begin{array}{c} X_{N}\left(S\right)=\left\{u\in P_{N}:u\left(\pm 1\right)=0\right\}\;\mathrm{for}\;S\neq 0,\;X_{N}\left(0\right)=\left\{u\in P_{N}:u\left(1\right)=0\right\}. \end{array}$$

We apply the Legendre–Galerkin approximation to Equations in (107) with the weight function (x+1), which corresponds to the Jacobian in the polar coordinate transformation. The approximate solution $\left(u_{N},v_{N}\right)\in X_{N}\left(S\right)$ then satisfies

(111) $$\begin{array}{c} \begin{array}{cc} \; & \int_{I}\partial_{t}u_{N}\cdot \omega \left(x+1\right)dx=\int_{I}v_{N}\cdot \omega \left(x+1\right)dx,\;t_{n}<t<t_{n+1},\;n\geq 0,\\ \; & \int_{I}\left(\alpha_{0}+\left(\alpha_{1}-\frac{4\alpha_{2}}{R^{2}}{▵}_{x,S}\right)\partial_{t}\right)v_{N}\cdot \omega \left(x+1\right)dx\\ \; & =\int_{I}\frac{2}{R^{2}}{▵}_{x,S}u_{N}\cdot \omega \left(x+1\right)dx+\int_{I}I_{N}g\left(u\right)\cdot \omega \left(x+1\right)dx+\mu \left(u_{N}\left(t_{n}\right)\right)\int_{I}u_{N}\left(t_{n}\right)\cdot \omega \left(x+1\right)dx,\\ \; & t_{n}<t<t_{n+1},\;n\geq 0,\\ \; & u_{N}\left(t_{n+1}\right):=\frac{u_{N}\left(t_{n+1}^{+}\right)}{∥u_{N}\left(t_{n+1}^{+}\right){∥}_{L^{2}\left(I\right)}},\;n\geq 0,\;\forall \omega \in X_{N}\left(S\right), \end{array} \end{array}$$

where $g\left(u\right)=\left(-gu^{2}+\tilde{H}\right)u$ and $I_{N}f$ is the interpolation of *f* in $P_{N}$ at the Legendre-Gauss–Lobotta collocation points.

To satisfy boundary conditions, we construct distinct function spaces for cases of S=0 and S≠0, respectively. For S≠0, we define the approximation function space

(112) $$\begin{array}{c} X_{N}\left(S\right)=span\left\{\chi_{i}\left(x\right)=L_{i}\left(x\right)-L_{i+2}\left(x\right),\;i=0,\cdots ,N-2\right\}, \end{array}$$

where $L_{i}\left(x\right)$ is the *i* th degree Legendre polynomial. Set

(113) $$\begin{array}{c} \begin{array}{cc} \; & a_{ij}=\int_{I}\chi_{j}^{\prime }\chi_{i}^{\prime }\left(x+1\right)dx,\;A={\left(a_{ij}\right)}_{i,j=0,1,\ldots ,N-2},\\ \; & b_{ij}=\int_{I}\frac{1}{x+1}\chi_{j}\chi_{i}dx,\;B={\left(b_{ij}\right)}_{i,j=0,1,\ldots ,N-2},\\ \; & c_{ij}=\int_{I}\chi_{j}\chi_{i}\left(x+1\right)dx,\;C={\left(c_{ij}\right)}_{i,j=0,1,\ldots ,N-2},\\ \; & u_{N}=\sum_{i=0}^{N-2}{\hat{u}}_{i}\chi_{i}\left(x\right),\;\vec{\hat{u}}={\left({\hat{u}}_{0},\cdots ,{\hat{u}}_{N-2}\right)}^{T},\\ \; & v_{N}=\sum_{i=0}^{N-2}{\hat{v}}_{i}\chi_{i}\left(x\right),\;\vec{\hat{v}}={\left({\hat{v}}_{0},\cdots ,{\hat{v}}_{N-2}\right)}^{T}\\ \; & \left(I_{N}g\left(u\right)\right)\left(x\right)=\sum_{i=0}^{N-2}{\hat{g}}_{i}\chi_{i}\left(x\right),\;\vec{\hat{g}}={\left({\hat{g}}_{0},\cdots ,{\hat{g}}_{N-2}\right)}^{T}. \end{array} \end{array}$$

The following results follow from the orthogonality of the Legendre polynomials. Matrix A and B are symmetric tri-diagonal, taking the following forms:

(114) $$a_{ij}=\left\{\begin{array}{cc} 2i+4, & j=i+1,\\ 4i+6, & j=i, \end{array}\right.\;b_{ij}=\left\{\begin{array}{cc} -\frac{2}{i+2}, & j=i+1,\\ \frac{2(2i+3)}{\left(i+1)(i+2\right)}, & j=i. \end{array}\right.$$

Matrix C is symmetric and seven-diagonal with

(115) $$\begin{array}{c} c_{ij}=\left\{\begin{array}{cc} -\frac{2(i+3)}{\left(2i+5)(2i+7\right)}, & j=i+3,\\ -\frac{2}{2i+5}, & j=i+2,\\ \frac{2}{\left(2i+1)(2i+5\right)}+\frac{2(i+3)}{\left(2i+5)(2i+7\right)}, & j=i+1,\\ \frac{2}{2i+1}+\frac{2}{2i+5}, & j=i. \end{array}\right. \end{array}$$

For S=0, we define the approximation function space,

(116) $$\begin{array}{c} X_{N}\left(0\right)=span\left\{\chi_{i}\left(x\right)=L_{i}\left(x\right)-L_{i+1}\left(x\right),\;i=0,\cdots ,N-1\right\}, \end{array}$$

and set

(117) $$\begin{array}{c} \begin{array}{cc} \; & a_{ij}=\int_{I}\chi_{j}^{\prime }\chi_{i}^{\prime }\left(x+1\right)dx,\;A={\left(a_{ij}\right)}_{i,j=0,1,\ldots ,N-1},\\ \; & b_{ij}=0,\;\forall \;i,j=0,1,\ldots ,N-1,\;B=O,\\ \; & c_{ij}=\int_{I}\chi_{j}\chi_{i}\left(x+1\right)dx,\;C={\left(c_{ij}\right)}_{i,j=0,1,\ldots ,N-1},\\ \; & u_{N}=\sum_{i=0}^{N-1}{\hat{u}}_{i}\chi_{i}\left(x\right),\;\vec{\hat{u}}={\left({\hat{u}}_{0},\cdots ,{\hat{u}}_{N-1}\right)}^{T},\\ \; & v_{N}=\sum_{i=0}^{N-1}{\hat{v}}_{i}\chi_{i}\left(x\right),\;\vec{\hat{v}}={\left({\hat{v}}_{0},\cdots ,{\hat{v}}_{N-1}\right)}^{T},\\ \; & \left(I_{N}g\left(u\right)\right)\left(x\right)=\sum_{i=0}^{N-1}{\hat{g}}_{i}\chi_{i}\left(x\right),\;\vec{\hat{g}}={\left({\hat{g}}_{0},\cdots ,{\hat{g}}_{N-1}\right)}^{T}. \end{array} \end{array}$$

Then we conclude that Matrix A is diagonal with

(118) $$\begin{array}{c} a_{ii}=2i+2. \end{array}$$

Matrix C is symmetric and penta-diagonal with

(119) $$\begin{array}{c} c_{ij}=\left\{\begin{array}{cc} -\frac{2(i+2)}{\left(2i+3)(2i+5\right)},\; & j=i+2,\\ \frac{4}{\left(2i+1)(2i+3)(2i+5\right)}, & j=i+1,\\ \frac{4(i+1)}{\left(2i+1)(2i+3\right)}, & j=i. \end{array}\right. \end{array}$$

We define

(120) $$\begin{array}{c} \begin{array}{cc} \; & u_{N}=\sum_{i=0}^{N_{1}}{\hat{u}}_{i}\chi_{i}\left(x\right),\;v_{N}=\sum_{i=0}^{N_{1}}{\hat{v}}_{i}\chi_{i}\left(x\right),\;\left(I_{N}g\left(u\right)\right)\left(x\right)=\sum_{i=0}^{N1}{\hat{g}}_{i}\chi_{i}\left(x\right),\\ \; & \vec{\hat{u}}={\left({\hat{u}}_{0},\cdots ,{\hat{u}}_{N_{1}}\right)}^{T},\;\vec{\hat{v}}={\left({\hat{v}}_{0},\cdots ,{\hat{v}}_{N_{1}}\right)}^{T},\;\vec{\hat{g}}={\left({\hat{g}}_{0},\cdots ,{\hat{g}}_{N1}\right)}^{T},\\ \; & \mathrm{with}\;N1=N-2\;\mathrm{when}\;S\neq 0,\;N1=N-1\;\mathrm{when}\;S=0. \end{array} \end{array}$$

Under the substitution $\omega =\chi_{i}\left(x\right)$, Equation (111) becomes

(121) $$\begin{array}{c} \begin{array}{cc} \; & \partial_{t}\vec{\hat{u}}=\vec{\hat{v}},\\ \; & \left[\left(\alpha_{0}+\alpha_{1}\partial_{t}\right)C+\frac{4\alpha_{2}}{R^{2}}\left(A+S^{2}B\right)\partial_{t}\right]\vec{\hat{v}}=-\frac{2}{R^{2}}\left(A+S^{2}B\right)\vec{\hat{u}}+C\left(\vec{\hat{g}}+\mu \left(u_{N}^{n}\right){\vec{\hat{u}}}^{n}\right),\\ \; & u_{N}\left(t_{n+1}\right):=\frac{u_{N}\left(t_{n+1}^{+}\right)}{∥u_{N}\left(t_{n+1}^{+}\right){∥}_{L^{2}\left(I\right)}},\;n\geq 0. \end{array} \end{array}$$

We now solve the magnetic field $\tilde{H}$ using the Legendre–Galerkin method as well. Employing coordinate transformation, Equation (15) with boundary conditions becomes

(122) $$\begin{array}{c} \begin{array}{cc} -\frac{4}{R^{2}}\left(\frac{d^{2}\tilde{H}}{dx^{2}}+\frac{1}{x+1}\frac{d\tilde{H}}{dx}\right) & =\gamma u^{2},\;x\in \left[-1,1\right],\\ \frac{d\tilde{H}}{dx}{|}_{x=-1} & =0,\;\frac{d\tilde{H}}{dx}{|}_{x=1}=\frac{\tilde{H}\left(1\right)}{2\ln(R)}. \end{array} \end{array}$$

An approximation of $\tilde{H}$ is sought within the space,

(123) $$\begin{array}{c} Y_{N}=\left\{\tilde{H}\in P_{N}:\frac{d\tilde{H}}{dx}{|}_{x=-1}=0,\;\frac{d\tilde{H}}{dx}{|}_{x=1}-\frac{\tilde{H}\left(1\right)}{2\ln(R)}=0\right\}. \end{array}$$

The basis functions are given by

(124) $$\begin{array}{c} \zeta_{i}\left(x\right)=L_{i}\left(x\right)+a_{i}L_{i+1}\left(x\right)+b_{i}L_{i+2}\left(x\right),\;i=0,1,\cdots ,N-1, \end{array}$$

where $a_{i}$ and $b_{i}$ are such that $\zeta_{i}\left(x\right)$ satisfies the boundary conditions of the function in $Y_{N}$. Solving the linear system yields

(125) $$\begin{array}{cc} \; & a_{i}=\frac{2i+3}{{\left(i+2\right)}^{2}\left(\ln(R)(i+1)(i+3)-1\right)},\;b_{i}=\frac{{\left(i+1\right)}^{2}}{{\left(i+2\right)}^{2}}\cdot \frac{\ln(R)\cdot i(i+2)-1}{1-\ln(R)(i+1)(i+3)},\\ & i=0,1,\cdots ,N-1. \end{array}$$

The Legendre–Galerkin approximation to Equation (122) is equivalent to finding ${\tilde{H}}_{N}=\sum_{j=0}^{N-2}{\hat{\tilde{H}}}_{j}\zeta_{j}\left(x\right)\in Y_{N}$ such that

(126) $$\begin{array}{c} -\frac{4}{R^{2}}\sum_{j=0}^{N-2}\int_{-1}^{1}\left(\frac{d^{2}\zeta_{j}}{dx^{2}}+\frac{1}{x+1}\frac{d\zeta_{j}}{dx}\right)\zeta_{i}\left(x\right)\left(x+1\right)dx{\hat{\tilde{H}}}_{j}=\int_{-1}^{1}I_{N}\left(\gamma u^{2}\right)\zeta_{i}\left(x\right)\left(x+1\right)dx. \end{array}$$

Let

(127) $$\begin{array}{c} h_{i,j}=\int_{-1}^{1}\left(\frac{d^{2}\zeta_{j}}{dx^{2}}+\frac{1}{x+1}\frac{d\zeta_{j}}{dx}\right)\;\zeta_{i}\left(x\right)\left(x+1\right)dx,\;H_{P}={\left(h_{ij}\right)}_{i,j=0,1,\ldots ,N-1}. \end{array}$$

We deduce that matrix $H_{P}$ is symmetric and tri-diagonal with

(128) $$\begin{array}{c} h_{ij}=\left\{\begin{array}{cc} 2\left(i+2\right)b_{i},\; & j=i+1,\\ 2\left(i+1\right)a_{i}+2\left(2i+3\right)b_{i}+2\left(i+2\right)a_{i}b_{i}, & j=i. \end{array}\right. \end{array}$$

We can now apply the two schemes ASNGF-I/II to Equation (121) to obtain two fully discrete schemes.

#### 2.1.3. Numerical Results for Central Vortex States

**Example** **1.**  *We present a systematic numerical study to identify practical working choices of $\alpha_{i}$ (i=0,1,2), guided by the strategy discussed in the previous sections. The parameter choices identified below should be understood as favorable choices within the finite sets of parameters tested, rather than as globally optimal values.*

*We compare our ASNGF-I/II schemes with existing alternatives. These include the GFLM-I/II scheme, the GFDN schemes (GFDN_BE and GFDN_BF) [7], the DSFDN schemes (DSFDN_LF and DSFDN_SI) [57], the semi-implicit DSFAL scheme (DSFAL_SI) [57], and the conventional PCG method [31]. Throughout this paper, all computations were carried out on a ThinkPad P15V Gen3 with an Intel i7-12700H CPU (14 physical cores, 20 threads, Lenovo Group Limited, Beijing, China).*

*We use the initial conditions $\phi_{S}^{0}\left(r\right)=\frac{1}{\sqrt{\pi S!}}r^{S}e^{-r^{2}/2}$ and ${\dot{\phi }}_{S}^{0}=0$, with error tolerance $\varepsilon =10^{-10}$ and stabilization parameter $\alpha =\max\{-\frac{1}{2}\left(-g\right|u^{n}{|}^{2}+{\tilde{H}}^{n}+\mu \left(u^{n}\right)),0\}$. For S=2, g=−30, γ=π, and τ=1, the computational domain is U=[0,20], and the dimension of the discrete Legendre space is $N_{l}=200$. The reference values are $E_{c}^{\mathrm{exa}}=0.4666956705613837$ and $\mu_{c}^{\mathrm{exa}}=0.5688732597658914$. The numerical results $E_{c}$ and $\mu_{c}$ denote the computed energy and chemical potential for different parameter choices, while #iter denotes the number of iterations (time steps) required to reach the prescribed convergence criterion. The MATLAB implementation of the ASNGF-I scheme is provided in Appendix A.1.*

*The leading $O(\tau^{-2})$ coefficient in ASNGF-I is $\alpha_{1}C+\frac{4\alpha_{2}}{R^{2}}\left(A+S^{2}B\right)$. Numerical experiments with $\alpha_{1}=\alpha_{2}=C_{1}\tau^{2}$, where $C_{1}$ is a constant, show that choices with $C_{1}=O\left(1\right)$ lead to relatively small iteration counts among the tested values, as shown in Table 2.*

*To elucidate the role of the Laplacian in the inertia term, we compute central vortex states using ASNGF-I with different values of $\alpha_{2}$. Table 3 shows that (i) the iteration count decreases substantially when the Laplacian term is included, compared with the tested case without it, and (ii) among the tested values of $\alpha_{2}$, the smallest iteration count is obtained at $\alpha_{2}=\alpha_{1}$. Parameterizing $\alpha_{0}^{n}=\left(C_{1}/t_{n}\right)\alpha_{1}$, where $t_{n}=n\tau$, Table 4 shows that $C_{1}=5$ gives the smallest iteration count among the tested values for ASNGF-I.*

*For ASNGF-II, Table 5 indicates that smaller values of $\alpha_{0}$ lead to faster convergence within the tested parameter range, with the most favorable performance observed as $\alpha_{0}$ approaches zero. Furthermore, Table 6 shows that choices of $C_{1}=O\left(10^{-1}\right)$ yield relatively small iteration counts among the tested values.*

*Table 7 provides two further observations for ASNGF-II: (i) convergence is not observed for the tested configuration in which the Laplacian term is excluded, indicating the important role of this term in the present numerical setting and (ii) among the tested values, the smallest iteration count is obtained at $\alpha_{2}=\alpha_{1}$.*

*Table 8 compares the numerical performance for a set of selected time steps τ. We choose $\alpha_{1}=\alpha_{2}=\tau^{2}$ and $\alpha_{0}^{n}=5\alpha_{1}/t_{n}$ for ASNGF-I, and $\alpha_{1}=\alpha_{2}=0.1\tau^{2}$ and $\alpha_{0}=0$ for ASNGF-II. For these particular parameter scalings, both ASNGF variants maintain nearly consistent iteration counts over the tested values of τ and converge to the prescribed central vortex state even for the extreme tested values $\tau =10^{\pm 60}$. Thus, for these parameter choices, the numerical results indicate that the explicit dependence on τ is effectively removed from the iteration behavior over the tested range. For other parameter choices, however, τ remains an effective algorithmic parameter.*

*To further illustrate the convergence behavior of ASNGF in this example, Figure 1 shows the evolution of the energy with respect to the iteration number for S=2, g=−30, and γ=π. The energy decreases rapidly during the initial iterations and subsequently approaches the reference value $E_{c}^{\mathrm{exa}}$.*

*The rapid and regular energy decay observed in Figure 1 provides a representative example of the convergence behavior of ASNGF for this relatively simple central-vortex problem. As shown later in Section 4, substantially different convergence behavior can occur for strongly non-convex multi-vortex problems. Thus, the convergence performance of ASNGF is problem-dependent.*

*As shown in Table 8, for the parameter settings and methods tested here, ASNGF-I and ASNGF-II require substantially fewer iterations than GFLM-I, GFDN_BE, and DSFDN_SI in this example. GFDN_FE and DSFDN_LF converge only for the tested time steps of order $O(10^{-3})$ or smaller and require considerably more iterations, while GFLM-II and DSFAL_SI do not converge for the tested time steps down to $O(10^{-5})$. In contrast, the PCG method, which is not based on gradient-flow dynamics, gives the smallest iteration count among the methods tested for this central vortex problem.*

*Similar trends are observed for the other tested winding numbers S. For example, for S=6, g=−100, γ=2π, and computational domain U=[0,40] with $N_{l}=400$, the reference values are $E_{c}^{\mathrm{exa}}=1.1385375982156505$ and $\mu_{c}^{\mathrm{exa}}=1.2964095192546976$. The results in Table 9 show behavior consistent with that observed in Table 8, providing additional evidence for the accuracy and computational efficiency of the ASNGF schemes for the central-vortex problems tested here.*

**Remark** **3.**  *The parameter study in Example 1 is intended to illustrate the influence of the coefficients and to identify representative parameter choices for the numerical experiments considered in this work. The resulting choices should not be interpreted as universally optimal parameters for ASNGF. In general, the convergence behavior can depend on the underlying physical problem, the computational domain, and the stage of the iteration. A systematic study of adaptive or problem-dependent parameter selection is beyond the scope of the present work and will be investigated separately.*

## 3. The Logarithmic Rotating GPE

The formation of self-bound droplets is a well-known macroscopic phenomenon in BEC systems. Recent experiments with ultracold bosonic quantum gases [58] have revealed a novel quantum liquid phase: ultra-dilute, self-bound droplets of BECs. These droplets possess densities that are orders of magnitude lower than air. As these droplets form from BECs, it is reasonable to assume they exhibit superfluid properties. A hallmark characteristic of superfluids is their distinct response to rotation, particularly the emergence of quantized vortices. Utilizing the leading-order correction to the mean-field energy, known as the Lee–Huang–Yang correction [59], a rotating binary BEC droplet is accurately described by a logarithmic GPE, incorporating angular momentum rotation as follows

(129) $$\begin{array}{c} i\partial_{t}\psi =-\frac{1}{2}\Delta \psi +V\psi +{\left|\psi \right|}^{2}\ln\left({\left|\psi \right|}^{2}\right)\psi -\Omega L_{z}\psi ,\;x\in R^{2},\; \end{array}$$

where the external potential is

(130) $$\begin{array}{c} V\left(x\right)=\frac{\gamma^{2}}{2}{\left|x\right|}^{2}+V_{0}e^{-\gamma_{0}{\left|x\right|}^{2}},\;\gamma ,\gamma_{0}>0,\;and\;V_{0}\geq 0, \end{array}$$

and the angular momentum operator $L_{z}=i\left(y\partial_{x}-x\partial_{y}\right)$.

Two important invariants of (129) are the mass (or normalization) of the wave function

(131) $$\begin{array}{c} N\left(\psi \left(\cdot ,t\right)\right)={∥\psi \left(\cdot ,t\right)∥}_{L^{2}\left(R^{2}\right)}^{2}\equiv \Xi ,\;t\geq 0, \end{array}$$

and the energy per particle

(132) $$\begin{array}{c} E\left(\psi \left(\cdot ,t\right)\right)=\int_{R^{2}}\frac{1}{2}{\left|\nabla \psi \right|}^{2}dx+\int_{R^{2}}V\left(x\right){\left|\psi \right|}^{2}dx+\int_{R^{2}}F\left(\psi \right)dx-\Omega \int_{R^{2}}\bar{\psi }L_{z}\psi dx\equiv E\left(\psi \left(\cdot ,0\right)\right), \end{array}$$

where

(133) $$\begin{array}{c} {F(|\psi |}^{2})=\frac{1}{2}{\left|\psi \left(t,x\right)\right|}^{4}\left({\ln|\psi \left(t,x\right)|}^{2}-\frac{1}{2}\right). \end{array}$$

By rescaling $\psi \to \sqrt{\Xi }\psi$, the wave function ψ is normalized by ${∥\psi \left(\cdot ,t\right)∥}_{L^{2}\left(R^{2}\right)}^{2}=1,\;t\geq 0.$ Consequently, (129) is transformed into

(134) $$\begin{array}{c} i\partial_{t}\psi =-\frac{1}{2}{\Delta \psi +V\psi +\Xi (\ln\left(\Xi \right)+\ln|\psi |}^{2}{)|\psi |}^{2}\psi -\Omega L_{z}\psi ,\;x\in R^{2}. \end{array}$$

One of the most important questions here is the calculation of the ground state, characterized by the following minimization problem, Find

$$\phi_{g}\in S:=\left\{\phi \in H_{0}^{1}\left(R^{2}\right)|{∥\phi ∥}^{2}=\Xi ,E\left(\phi \right)<\infty \right\}$$

such that

(135) $$\begin{array}{c} E_{g}:=E\left(\phi_{g}\right)=\min_{\phi \in S}E\left(\phi \right). \end{array}$$

**Remark** **4.**  *In [60], it was shown that, in Hilbert space $X:=\{\psi \in H^{1}\left(R^{2}\right)|x\to |x|\psi \left(x\right)\in L^{2}\left(R^{2}\right)\}$, there exists a ground state $\phi_{g}$ of* (132) *when Ω<γ; when Ω>γ, there exists no ground state.*

Due to the blowup of the logarithmic nonlinearity, i.e., lnρ→−∞ as $\rho \to 0^{+}$, where $\rho ={\left|\psi \right|}^{2}$ denotes the density, it is a challenge to design numerical methods for resolving the ground state. To circumvent this singularity and suppress roundoff errors, we adopt the regularized scheme introduced in [61], which employs a small regularization parameter 0<ε≪1 and a polynomial of degree *n*. Under this regularization, the Euler–Lagrange equation is approximated by

(136) $$\begin{array}{c} \mu \phi^{\varepsilon }=-\frac{1}{2}\Delta \phi^{\varepsilon }+V\phi^{\varepsilon }+f_{n}^{\varepsilon }\left(|\phi^{\varepsilon }{|}^{2}\right)\phi^{\varepsilon }-\Omega L_{z}\phi^{\varepsilon }.\; \end{array}$$

where

(137) $$\begin{array}{c} f_{n}^{\varepsilon }\left(\rho \right)=\rho \ln\rho \chi_{\left\{\rho \geq \varepsilon^{2}\right\}}+q_{n+1}^{\varepsilon }\left(\rho \right)\chi_{\left\{\rho <\varepsilon^{2}\right\}},\;\rho \geq 0, \end{array}$$

(138) $$\begin{array}{c} q_{n+1}^{\varepsilon }\left(\rho \right)=\rho \ln\left(\varepsilon^{2}\right)-\rho \sum_{k=1}^{n}\frac{1}{k}{\left(1-\frac{\rho }{\varepsilon^{2}}\right)}^{k}-\frac{1}{2}\rho {\left(1-\frac{\rho }{\varepsilon^{2}}\right)}^{n},\; \end{array}$$

and $\chi_{A}$ denotes the characteristic function of set *A*, subject to constraint

(139) $$\begin{array}{c} ∥\phi^{\varepsilon }{∥}_{L^{2}\left(R^{2}\right)}^{2}=\int_{R^{2}}{\left|\phi^{\varepsilon }\left(x\right)\right|}^{2}dx=\Xi . \end{array}$$

We numerically compute the ground state for the logarithmic rotating GPE. In the ASNGF-I scheme, only the Laplacian term $-\frac{1}{2}\Delta \phi^{\varepsilon }$ in Equation (136) is treated implicitly, and all other terms, including the angular momentum rotation term $-\Omega L_{z}$, are evaluated explicitly at the previous iteration. The stabilization parameter α is chosen as

(140) $$\begin{array}{c} \alpha =\frac{1}{2}\max\left(V\left(x\right)+f_{n}^{\varepsilon }\left(\right|\phi_{S}^{n}{|}^{2})+\Omega \pi M-\mu^{n},\;0\right), \end{array}$$

where *M* is the number of grid points in each direction. With this treatment, the implicit operator at each iteration takes the form $\frac{\alpha_{0}}{\tau }+\frac{\alpha_{1}-\alpha_{2}\Delta }{\tau^{2}}+\alpha -\frac{1}{2}\Delta$, which is independent of the rotation term $-\Omega L_{z}$. This choice is essential for efficiency, as implicit $L_{z}$ treatment would couple spatial directions and increase the solving cost.

When external potential V(x) satisfies $\lim_{|x|\to \infty }V\left(x\right)=\infty$, function $\phi^{\varepsilon }$ exhibits smoothness and exponential far-field decay. In the numerical experiments presented below, all algorithms are performed over the bounded domain U = [−16,16] using the spatial Fourier-pseudospectral discretization with the dimension of the discrete Fourier space $N_{f}=256$. The initial value is chosen as $\phi^{\varepsilon ,0}=\sqrt{\Xi }\frac{{\tilde{\phi }}^{\varepsilon ,0}}{∥{\tilde{\phi }}^{\varepsilon ,0}∥}$ and ${\dot{\phi }}^{\varepsilon ,0}=0$, where ${\tilde{\phi }}^{\varepsilon ,0}=\frac{1-\Omega }{\sqrt{\pi }}e^{-\frac{x^{2}+y^{2}}{2}}+\frac{\Omega (x+iy)}{\sqrt{\pi }}e^{-\frac{x^{2}+y^{2}}{2}}$. Throughout the simulations, $\gamma =\gamma_{0}=V_{0}=1$ is used. The stopping criterion requires the maximum residual (measured at the grid points) of the Euler–Lagrange Equation (136) to fall below $10^{-10}$.

The effects of regularization parameter ε and polynomial degree *n* on ground states are first investigated. Using ASNGF-II with parameters Ω=0.9,Ξ=10,ε=0.001,n=100,$\tau =1,\alpha_{1}=100\tau ,\alpha_{2}=10\tau ,\alpha_{0}=0$ yields reference energy $E^{exa}=13.341225274544906$ and Lagrange multiplier $\mu^{exa}=1.145138024694604$. Table 10 and Table 11 compare ground-state energy *E*, chemical potential μ, and iteration count #iter for varying ε (n=100 fixed) and varying *n* (ε=0.001 fixed), respectively. Table 10 shows convergence data of both energy and chemical potential as ε→0, while iteration counts exhibit minimal sensitivity to variations in ε. Table 11 shows that fifth-order polynomial approximation (n=5) yields accurate results for both the ground-state energy and the chemical potential.

We then study how $\alpha_{0}$, $\alpha_{1}$, and $\alpha_{2}$ affect the ground-state energy, the chemical potential, and the number of iterations in ASNGF-I/II, following Section 2’s methodology. For ASNGF-I, with $\alpha_{1}=\alpha_{2}=C_{1}\tau^{2}$, convergence requires $C_{1}\geq O\left(1\right)$. The best convergence occurs when $\alpha_{1}$ and $\alpha_{2}$ are comparable in magnitude, whereas the choice of $\alpha_{0}$ (0 or >3) has minimal impact on the number of iterations. For ASNGF-II, the fastest convergence arises only when $\alpha_{0}=0$.

Table 12 compares the performance of the ground-state computation with respect to various numerical schemes. The ASNGF scheme demonstrates significant acceleration over GFDN [7], GFLM [18], DSFDN and DSFAL [57] methods in both the speed of convergence and accuracy. Most importantly, we note that the ASNGF’s convergence rate exhibits exceptional time-step independence, achieving rapid convergence to ground states even at extreme time steps ($\tau =10^{\pm 60}$).

## 4. The Rapid Rotating GPE

Gaseous BECs constitute exceptional platforms for studying superfluidity, where quantized vortices play fundamental roles. Recent experimental advances [62,63] enable engineering of confinement potentials beyond harmonic traps—including optical lattices—facilitating novel approaches to probe BEC properties with and without time-varying potentials. These developments have spurred considerable research focus on vortex lattice configurations in rapidly rotating BECs with strong repulsive interactions. In this section, we consider a two-dimensional rapidly rotating BEC system confined in a radial trap, with the energy functional given by

(141) $$\begin{array}{c} E\left(\phi \right)=\int_{R^{2}}\left[\frac{1}{2}{\left|\nabla \phi \right|}^{2}+{V\left(x\right)|\phi |}^{2}+\frac{g}{2}{\left|\phi \right|}^{4}-\Omega \phi^{*}L_{z}\phi \right]dx, \end{array}$$

subject to normalization

(142) $$\begin{array}{c} N\left(\phi \right)={∥\phi ∥}_{L^{2}\left(R^{2}\right)}^{2}\equiv 1, \end{array}$$

where $V\left(x\right)=\left(1-\chi \right)\left(x^{2}+y^{2}\right)+\frac{\kappa {\left(x^{2}+y^{2}\right)}^{2}}{4}$ is a harmonic-plus-optical lattice potential, *g* is the nonlinearity strength, Ω is the rotation speed, and $L_{z}=i\left(y\partial_{x}-x\partial_{y}\right)$ is the angular momentum operator.

To compute low-energy stationary states and ground-state candidates of the rotating BEC, we apply the ASNGF methods for temporal discretization and the Fourier pseudospectral method for spatial discretization. We focus on the ASNGF schemes in this section, since the conventional gradient-flow methods considered in the preceding sections become computationally expensive for these rapidly rotating two-dimensional problems. The computational domain and mesh size are chosen, respectively, as $U={\left[-10,10\right]}^{2}$ and $h=\frac{1}{16}$. Fixing χ=1.2 and κ=0.3, we take the initial data as $\phi^{0}\left(x\right)=\frac{1}{\sqrt{\pi }}e^{-(x^{2}+y^{2})/2},{\dot{\phi }}^{0}\left(x\right)=0.$ Since the initial state is cylindrically symmetric, the iteration may remain close to a nearly symmetric configuration for a prolonged period when the target rotation frequency is relatively small. This may delay the amplification of small symmetry-breaking perturbations arising from spatial discretization and round-off errors, which are required for the development of a non-axisymmetric vortex configuration.

For the case with target rotation frequency Ω=1, we therefore employ a symmetry-breaking initialization strategy: Ω=5 is used during the first 2000 iterations, after which it is reset to the target value Ω=1. The temporary increase in the rotation frequency facilitates the amplification of these perturbations and accelerates departure from the nearly cylindrically symmetric configuration. This procedure modifies only the initialization trajectory. All subsequent iterations and the convergence test are performed for the target stationary problem with Ω=1. We choose $\tau =1,\alpha_{1}=10\tau^{2},\alpha_{2}=\tau^{2},\alpha_{0}^{n}=\frac{5}{t_{n}}\alpha_{1}$ (where $t_{n}=n\tau$) for ASNGF-I and $\alpha_{0}=0,\alpha_{1}=\alpha_{2}=\tau$ for ASNGF-II. The stopping criterion is that the maximum residual (measured at the grid points) of the Euler–Lagrange equation

(143) $$\begin{array}{c} \mu \phi \left(x\right)=-\frac{1}{2}\Delta \phi \left(x\right)+V\left(x\right)\phi \left(x\right)+g{\left|\phi \left(x\right)\right|}^{2}\phi \left(x\right)-\Omega L_{z}\phi \left(x\right) \end{array}$$

is less than $10^{-6}$. We employ the same semi-implicit treatment for the ASNGF-I scheme as described in Section 3, where only the Laplacian term in Equation (143) is treated implicitly, while the angular momentum term $-\Omega L_{z}$ and all other terms are evaluated explicitly. The stabilization parameter is chosen as $\alpha =\frac{1}{2}\max(V+g|\phi^{n}{|}^{2}+\Omega \pi M-\mu^{n},0)$, where *M* is the number of grid points. The MATLAB implementation of the ASNGF-I scheme is provided in Appendix A.2.

The computed energies and computational costs for ASNGF-I and ASNGF-II are summarized in Table 13 for different values of *g* and Ω. The rapidly rotating multi-vortex problems considered here are characterized by highly non-convex energy landscapes with multiple competing vortex configurations. Consequently, the stationary configuration reached in a particular run may depend on the initial state, symmetry-breaking perturbations, computational domain, and algorithmic parameters.

To illustrate the convergence behavior in such a challenging case, we consider g=1000 and Ω=1. Figure 2 shows the energy difference $|E^{n}-E_{\mathrm{stat}}|$ on a logarithmic scale, where $E_{\mathrm{stat}}\approx 11.6586$ denotes the stationary energy approached by the present ASNGF trajectory. After the target rotation frequency is restored, the energy gradually approaches $E_{\mathrm{stat}}$. On a linear energy scale, the energy appears nearly constant during the later stage of the computation because its deviation from $E_{\mathrm{stat}}$ has already become very small. The logarithmic representation in Figure 2 resolves these small differences and shows the continued decay of $|E^{n}-E_{\mathrm{stat}}|$ over the extended iteration. In contrast, the central-vortex problem in Example 1 exhibits much more rapid convergence toward its reference energy; see Figure 1. This comparison illustrates the strongly problem-dependent convergence characteristics of ASNGF.

A lower-energy vortex configuration can nevertheless be obtained for the same problem, showing that the stationary configuration reached by the present ASNGF run is not a global minimizer. This observation is consistent with the glassy character of the vortex-lattice energy landscape, where multiple competing stationary configurations may coexist. Accordingly, the inertial mechanism should be understood as enhancing exploration of non-convex energy landscapes and, in some cases, facilitating escape from metastable regions, rather than as providing a guarantee of global optimization. The energies reported in Table 13 therefore correspond to stationary configurations obtained under the specified initial conditions, algorithmic parameters, computational domain, and numerical setting, and should not in general be interpreted as certified global minimum energies.

The corresponding density profiles $|\phi_{g}{\left(x\right)|}^{2}$ are shown in Figure 3. The computed stationary configurations exhibit distinct vortex structures as *g* and Ω vary. At relatively low rotation frequencies, multi-vortex lattice configurations are observed. As Ω increases, the centrifugal effect progressively depletes the condensate density near the center, leading to the formation of a pronounced central density hole. Further increases in Ω enlarge the central void and increase the vortex population while modifying the vortex-containing annular region through the competition between rotational and confinement effects.

## 5. Conclusions

In this work, we developed an accelerated, stabilizer-based normalized gradient flow (ASNGF) framework for computing stationary states of gradient-flow problems subject to a normalization constraint. The method incorporates an inertial mechanism into normalized gradient-flow dynamics and leads to two practical temporal discretizations, ASNGF-I and ASNGF-II. For the Gross–Pitaevskii equations considered in this work, the resulting schemes provide an alternative to conventional normalized gradient-flow methods for the computation of stationary states.

For the central-vortex problems studied in Section 2, the numerical experiments show that appropriate choices of the inertial and stabilization parameters can substantially reduce the iteration count compared with several conventional gradient-flow schemes tested in this work. The parameter studies also provide practical guidance for selecting $\alpha_{0}$, $\alpha_{1}$, and $\alpha_{2}$. These choices should, however, be understood as favorable parameter settings within the finite ranges examined numerically, rather than as globally optimal parameters. In particular, Example 1 exhibits a rapid and regular energy decay toward the reference stationary energy for the selected parameter setting.

The numerical experiments further demonstrate that the convergence behavior of ASNGF is problem- and parameter-dependent. This dependence is particularly evident for the rapidly rotating multi-vortex problems considered in Section 4. In contrast to the rapid and regular convergence observed in Example 1, vortex-lattice problems may exhibit substantially longer relaxation processes and greater sensitivity to symmetry breaking, computational domain, and algorithmic parameters. For a representative rapidly rotating multi-vortex case, extended computations approach a stationary state, while a lower-energy state can nevertheless be obtained for the same physical parameters. This observation highlights an important limitation of the method: although the inertial mechanism is intended to enhance exploration of non-convex energy landscapes and may facilitate escape from certain metastable regions, it does not guarantee escape from every higher-energy stationary state or convergence to a global minimizer.

Taken together, the numerical results indicate that ASNGF can provide substantial acceleration for suitable problems and parameter settings, while its effectiveness depends on the underlying energy landscape and the choice of algorithmic parameters. The linearized stability analysis and the parameter studies presented in this work provide useful guidance for practical parameter selection, but they do not constitute a general optimal-parameter theory. Developing adaptive parameter-selection, damping, and restart strategies and systematically investigating parameter and domain-size sensitivity, particularly for strongly rotating vortex-lattice problems, are therefore important directions for future work.

Several further questions remain open. First, extending ASNGF to three-dimensional problems is a natural direction. The substantially increased computational cost and more complicated energy landscapes in three dimensions provide an important setting in which to assess the effectiveness of inertial acceleration and stabilization. Such an extension would also require further analysis of the spectral and stability properties of the corresponding discrete operators. Second, the applicability of ASNGF to nonlinear eigenvalue problems beyond BECs, including nonlinear optical systems and other constrained variational problems, deserves investigation. Third, a more complete convergence theory for the fully discrete ASNGF schemes remains an important theoretical problem. The dissipation estimates and numerical results presented here support the proposed schemes under the stated conditions, while nonlinear stability, convergence to stationary points, and quantitative convergence rates require further study. Extensions to more general BEC models, including spin–orbit coupled systems and other interaction regimes, also provide interesting directions for future work.

Overall, ASNGF provides a flexible inertial normalized-gradient-flow framework for stationary-state computations. The results presented here demonstrate its potential for accelerating several classes of BEC problems, while also identifying limitations associated with non-convexity, parameter sensitivity, and competing stationary states. These observations provide a basis for further development of adaptive and more robust accelerated gradient-flow algorithms.

## Figures and Tables

**Figure 1:**
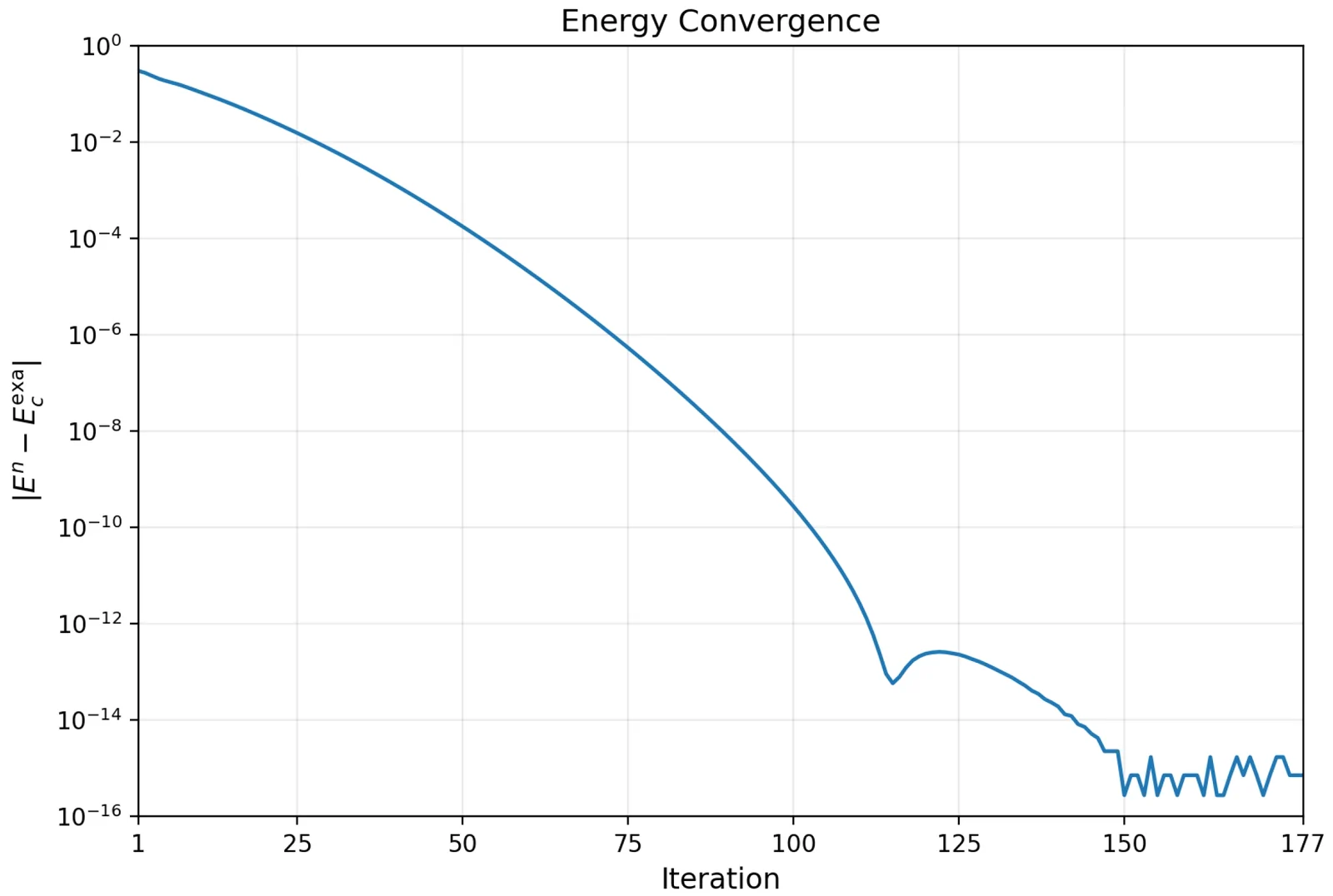
Evolution of the energy difference $|E^{n}-E_{c}^{\mathrm{exa}}|$ with respect to the iteration number for S=2, g=−30, and γ=π. The logarithmic vertical scale highlights the rapid decay toward the reference energy $E_{c}^{\mathrm{exa}}$ and the late-stage convergence behavior.

**Figure 2:**
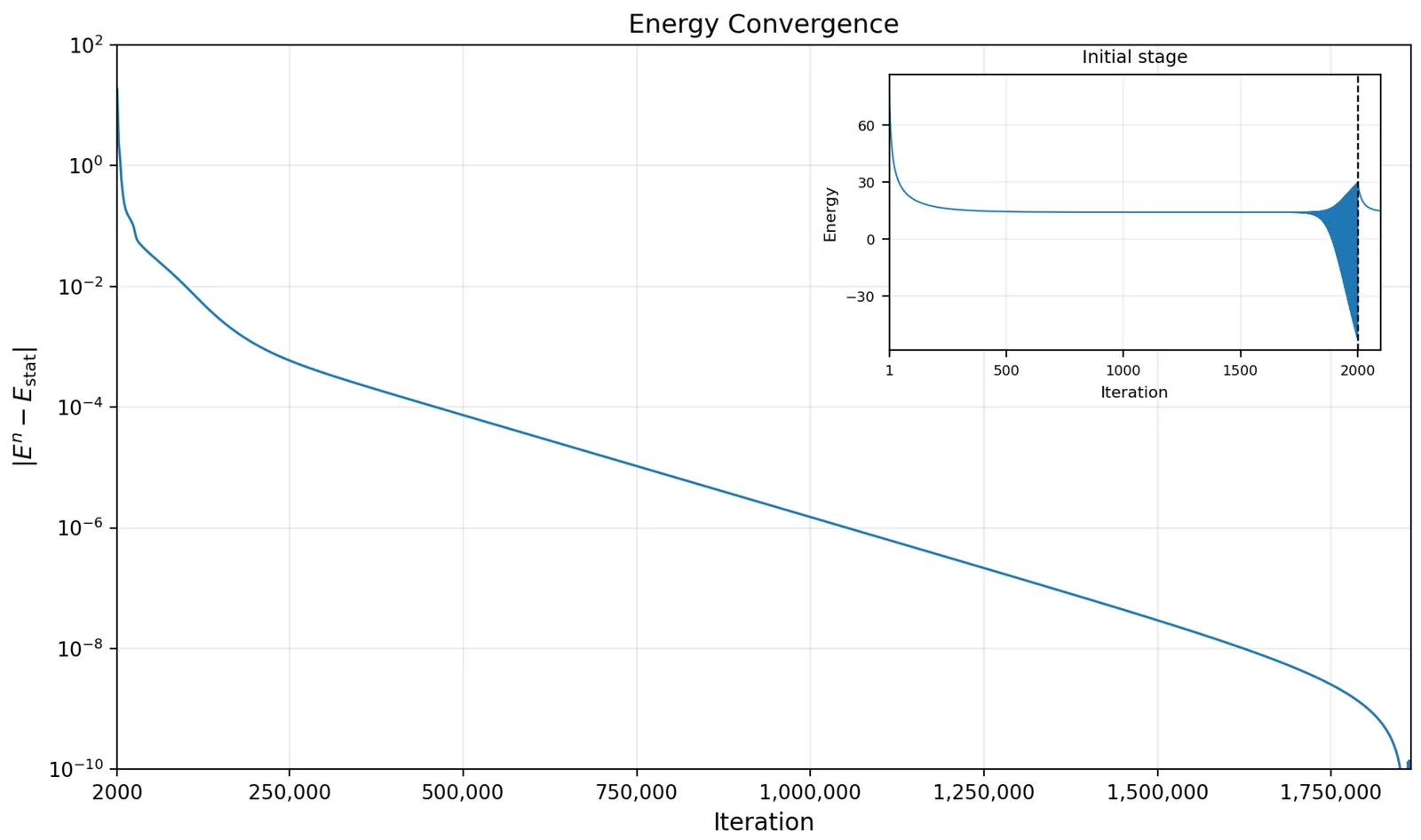
Evolution of the energy difference $|E^{n}-E_{\mathrm{stat}}|$ on a logarithmic scale for the rapidly rotating multi-vortex problem with g=1000 and Ω=1, where $E_{\mathrm{stat}}\approx 11.6586$ is the stationary energy approached by the present ASNGF trajectory. The inset shows the initial energy evolution, with Ω=5 used for the first 2000 iterations to facilitate symmetry breaking and the target value Ω=1 restored thereafter.

**Figure 3:**
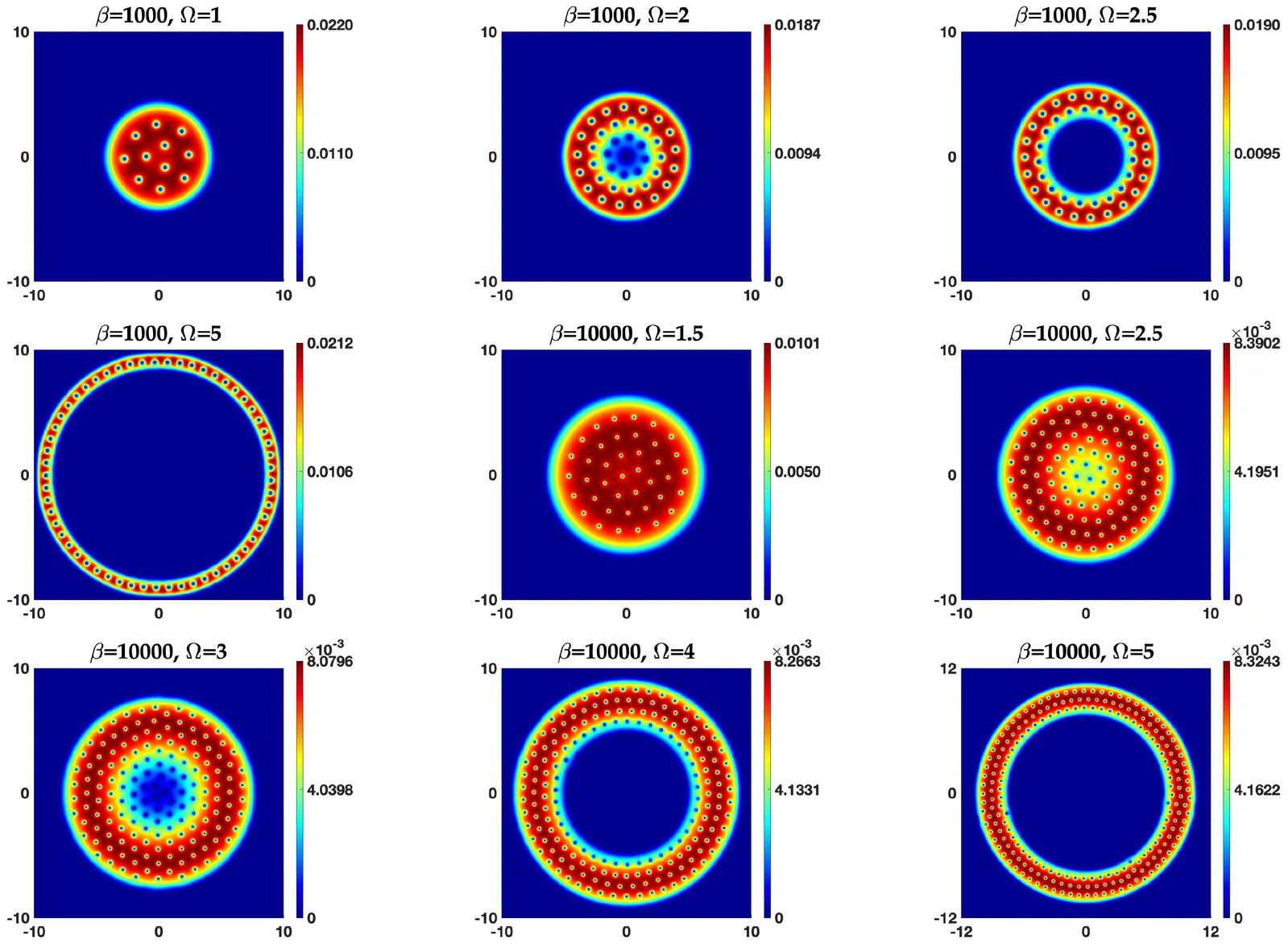
Corresponding contour plots of the density function $|\phi^{\varepsilon }{|}^{2}$.

**Table 1:** The value of $|g_{b}|$ vs. *S* reported in [52].

***S***	**0**	**1**	**2**	**3**	**4**	**5**	**6**	**7**
$|g_{b}|$	5.85	24.16	44.88	66.21	87.75	109.38	131.06	152.76
S	**8**	**9**	**10**	**11**	**12**	**13**	**14**	**15**
$|g_{b}|$	174.47	196.20	217.94	239.68	261.42	283.17	304.92	326.67

**Table 2:** Performance of the ASNGF-I scheme with $\alpha_{1}=\alpha_{2}=C_{1}\cdot \tau^{2},\alpha_{0}^{n}=\frac{5}{t_{n}}\alpha_{1}$.

$C_{1}$	CPU (s)	$|E_{c}-E_{c}^{\mathrm{exa}}|$	$|\mu_{c}-\mu_{c}^{\mathrm{exa}}|$	#iter
0.01	-	-	-	-
0.1	-	-	-	-
1	1.69	1.9 $\times \;10^{-15}$	3.4 $\times \;10^{-15}$	177
10	11.83	2.0 $\times \;10^{-15}$	4.8 $\times \;10^{-10}$	1130
100	100.87	8.1 $\times \;10^{-14}$	2.6 $\times \;10^{-10}$	9321

**Table 3:** Performance of the ASNGF-I scheme with $\alpha_{1}=\tau^{2},\alpha_{2}=C_{1}\cdot \alpha_{1},\alpha_{0}^{n}=\frac{5}{t_{n}}\alpha_{1}$.

$C_{1}$	CPU (s)	$|E_{c}-E_{c}^{\mathrm{exa}}|$	$|\mu_{c}-\mu_{c}^{\mathrm{exa}}|$	#iter
0	2.46	2.0 $\times \;10^{-16}$	2.2 $\times \;10^{-10}$	247
0.01	2.43	6.0 $\times \;10^{-16}$	2.0 $\times \;10^{-10}$	247
0.1	2.34	1.3 $\times \;10^{-15}$	2.1 $\times \;10^{-10}$	242
1	1.69	1.9 $\times \;10^{-15}$	3.4 $\times \;10^{-15}$	177
10	8.95	9.0 $\times \;10^{-16}$	1.6 $\times \;10^{-10}$	916

**Table 4:** Performance of the ASNGF-I scheme with $\alpha_{1}=\alpha_{2}=\tau^{2},\alpha_{0}^{n}=C_{1}\cdot \frac{\alpha_{1}}{t_{n}}$.

$C_{1}$	CPU (s)	$|E_{c}-E_{c}^{\mathrm{exa}}|$	$|\mu_{c}-\mu_{c}^{\mathrm{exa}}|$	#iter
0	3.89	1.0 $\times \;10^{-16}$	1.4 $\times \;10^{-10}$	402
1	2.78	1.1 $\times \;10^{-15}$	1.3 $\times \;10^{-10}$	284
3	1.83	2.0 $\times \;10^{-16}$	1.3 $\times \;10^{-10}$	186
5	1.69	1.9 $\times \;10^{-15}$	3.4 $\times \;10^{-15}$	177
7	1.88	3.0 $\times \;10^{-16}$	4.1 $\times \;10^{-10}$	193
9	1.98	6.0 $\times \;10^{-16}$	4.7 $\times \;10^{-10}$	202

**Table 5:** Performance of the ASNGF-II scheme with $\alpha_{1}=\alpha_{2}=\tau^{2},\alpha_{0}^{n}=C_{1}\cdot \frac{\alpha_{1}}{t_{n}}$.

$C_{1}$	CPU (s)	$|E_{c}-E_{c}^{\mathrm{exa}}|$	$|\mu_{c}-\mu_{c}^{\mathrm{exa}}|$	#iter
0	2.95	7.0 $\times \;10^{-16}$	3.3 $\times \;10^{-10}$	302
1 $\times \;10^{-10}$	3.03	9.0 $\times \;10^{-16}$	3.4 $\times \;10^{-10}$	303
1 $\times \;10^{-6}$	13.04	1.2 $\times \;10^{-15}$	2.9 $\times \;10^{-10}$	1328
1 $\times \;10^{-5}$	144.50	5.0 $\times \;10^{-16}$	2.8 $\times \;10^{-10}$	13,186

**Table 6:** Performance of the ASNGF-II scheme with $\alpha_{0}=0,\alpha_{1}=\alpha_{2}=C_{1}\cdot \tau^{2}$.

$C_{1}$	CPU (s)	$|E_{c}-E_{c}^{\mathrm{exa}}|$	$|\mu_{c}-\mu_{c}^{\mathrm{exa}}|$	#iter
0.001	5.15	1.3 $\times \;10^{-15}$	6.2 $\times \;10^{-10}$	532
0.01	4.47	1.4 $\times \;10^{-15}$	6.3 $\times \;10^{-10}$	459
0.1	1.85	1.3 $\times \;10^{-15}$	3.6 $\times \;10^{-10}$	190
1	2.95	7.0 $\times \;10^{-16}$	3.3 $\times \;10^{-10}$	302

**Table 7:** Performance of the ASNGF-II scheme with $\alpha_{0}=0,\alpha_{1}=0.1\cdot \tau^{2},\alpha_{2}=C_{1}\cdot \alpha_{1}$.

$C_{1}$	CPU (s)	$|E_{c}-E_{c}^{\mathrm{exa}}|$	$|\mu_{c}-\mu_{c}^{\mathrm{exa}}|$	#iter
0	-	-	-	-
0.01	85.14	1.9 $\times \;10^{-15}$	1.1 $\times \;10^{-10}$	7656
0.1	9.23	1.1 $\times \;10^{-15}$	3.8 $\times \;10^{-10}$	954
1	1.85	1.3 $\times \;10^{-15}$	3.6 $\times \;10^{-10}$	190
10	4.20	1.3 $\times \;10^{-16}$	2.6 $\times \;10^{-10}$	436

**Table 8:** Comparison of different methods at S=2, g=−30, γ=π.

Method	τ	CPU (s)	$|E_{c}-E_{c}^{\mathrm{exa}}|$	$|\mu_{c}-\mu_{c}^{\mathrm{exa}}|$	#iter
ASNGF-I	1 $\times \;10^{60}$	1.69	1.5 $\times \;10^{-15}$	2.8 $\times \;10^{-15}$	177
	1	1.69	1.9 $\times \;10^{-15}$	3.4 $\times \;10^{-15}$	177
	1 $\times \;10^{-60}$	1.69	3.0 $\times \;10^{-16}$	5.6 $\times \;10^{-16}$	177
GFLM-I	10	-	-	-	-
	1	5.70	7.0 $\times \;10^{-16}$	3.9 $\times \;10^{-10}$	590
	0.1	34.54	2.0 $\times \;10^{-16}$	4.3 $\times \;10^{-10}$	3488
	0.01	338.97	8.0 $\times \;10^{-16}$	4.4 $\times \;10^{-10}$	32,429
ASNGF-II	1 $\times \;10^{60}$	1.85	1.6 $\times \;10^{-15}$	2.6 $\times \;10^{-10}$	190
	1	1.85	1.3 $\times \;10^{-15}$	3.6 $\times \;10^{-10}$	190
	1 $\times \;10^{-60}$	1.85	2.4 $\times \;10^{-15}$	3.4 $\times \;10^{-10}$	190
GFLM-II	0.01	-	-	-	-
	1 $\times \;10^{-5}$	-	-	-	-
GFDN_BE	1	-	-	-	-
	0.1	186.90	7.0 $\times \;10^{-16}$	4.4 $\times \;10^{-10}$	3621
	0.01	1095.25	2.0 $\times \;10^{-16}$	4.3 $\times \;10^{-10}$	32,564
GFDN_BF	0.01	-	-	-	-
	0.001	2030.09	1.9 $\times \;10^{-7}$	6.4 $\times \;10^{-4}$	200,000
DSFDN_LF	0.01	-	-	-	-
	0.001	2261.76	4.4 $\times \;10^{-15}$	9.3 $\times \;10^{-8}$	200,000
DSFDN_SI	1	-	-	-	-
	0.1	88.85	1.6 $\times \;10^{-15}$	1.3 $\times \;10^{-10}$	8578
	0.01	614.52	4.0 $\times \;10^{-16}$	1.1 $\times \;10^{-10}$	54,152
DSFAL_SI	0.01	-	-	-	-
	1 $\times \;10^{-5}$	-	-	-	-
PCG	-	0.59	5.0 $\times \;10^{-16}$	1.4 $\times \;10^{-10}$	39

**Table 9:** Comparison of different methods at S=6, g=−100, γ=2π.

Method	τ	CPU (s)	$|E_{c}-E_{c}^{\mathrm{exa}}|$	$|\mu_{c}-\mu_{c}^{\mathrm{exa}}|$	#iter
ASNGF-I	1 $\times \;10^{60}$	413.03	5.0 $\times \;10^{-16}$	2.0 $\times \;10^{-16}$	2218
	1	411.10	4.0 $\times \;10^{-16}$	8.7 $\times \;10^{-14}$	2218
	1 $\times \;10^{-60}$	412.74	1.8 $\times \;10^{-15}$	1.3 $\times \;10^{-13}$	2218
GFLM-I	1	-	-	-	-
	0.1	2289.11	4.0 $\times \;10^{-16}$	4.8 $\times \;10^{-10}$	11,971
	0.01	18,694.70	3.6 $\times \;10^{-15}$	5.0 $\times \;10^{-10}$	99,368
ASNGF-II	1 $\times \;10^{60}$	1785.20	1.6 $\times \;10^{-15}$	1.7 $\times \;10^{-10}$	9610
	1	1785.91	4.6 $\times \;10^{-15}$	1.8 $\times \;10^{-10}$	9610
	1 $\times \;10^{-60}$	1798.87	2.2 $\times \;10^{-15}$	1.8 $\times \;10^{-10}$	9611
GFLM-II	0.01	-	-	-	-
	1 $\times \;10^{-5}$	-	-	-	-
GFDN_BE	0.1	-	-	-	-
	0.01	34,948.44	2.7 $\times \;10^{-8}$	4.4 $\times \;10^{-4}$	50,000
GFDN_BF	0.01	20,938.27	3.5 $\times \;10^{-4}$	5.5 $\times \;10^{-2}$	100,000
	0.001	20,954.13	1.2 $\times \;10^{-3}$	9.7 $\times \;10^{-2}$	100,000
DSFDN_LF	0.01	-	-	-	-
	0.001	-	-	-	-
DSFDN_SI	1	-	-	-	-
	0.1	19,211.12	1.1 $\times \;10^{-16}$	1.4 $\times \;10^{-9}$	100,000
	0.01	19,242.67	2.0 $\times \;10^{-16}$	1.6 $\times \;10^{-9}$	100,000
DSFAL_SI	0.01	-	-	-	-
	1 $\times \;10^{-5}$	-	-	-	-
PCG	-	28.78	4.0 $\times \;10^{-16}$	1.2 $\times \;10^{-9}$	100

**Table 10:** Comparison of different regularization parameter ε for energy *E*, chemical potential μ, and iteration count #iter.

ε	CPU (s)	$|E-E^{\mathrm{exa}}|$	$|\mu -\mu^{\mathrm{exa}}|$	#iter
0.01	1387.06	9.6 $\times \;10^{-13}$	2.2 $\times \;10^{-12}$	3976
0.05	1393.21	2.1 $\times \;10^{-9}$	2.2 $\times \;10^{-8}$	3976
0.1	1369.58	9.8 $\times \;10^{-8}$	1.0 $\times \;10^{-6}$	3970
0.2	1365.20	1.5 $\times \;10^{-5}$	4.9 $\times \;10^{-5}$	3751

**Table 11:** Comparison of different polynomial approximation degree *n* for energy *E*, chemical potential μ, and iteration count #iter.

*n*	CPU (s)	$|E-E_{c}^{\mathrm{exa}}|$	$|\mu -\mu^{\mathrm{exa}}|$	#iter
5	481.49	1.4 $\times \;10^{-14}$	1.1 $\times \;10^{-15}$	3976
10	525.84	5.3 $\times \;10^{-15}$	1.1 $\times \;10^{-15}$	3975

**Table 12:** Comparison of different methods for ground states of the logarithmic rotating GPE.

Method	τ	CPU (s)	$|E-E^{\mathrm{exa}}|$	$|\mu -\mu^{\mathrm{exa}}|$	#iter
ASNGF-I	1 $\times \;10^{60}$	34.36	1.0 $\times \;10^{-14}$	1.1 $\times \;10^{-15}$	4679
	1	34.87	5.4 $\times \;10^{-16}$	3.2 $\times \;10^{-16}$	4679
	1 $\times \;10^{-60}$	35.94	8.8 $\times \;10^{-15}$	6.2 $\times \;10^{-16}$	4679
GFLM-I	0.1	-	-	-	-
	0.01	879.28	1.5 $\times \;10^{-14}$	8.8 $\times \;10^{-16}$	114,329
	0.001	4302.10	2.8 $\times \;10^{-14}$	3.9 $\times \;10^{-15}$	564,673
ASNGF-II	1 $\times \;10^{60}$	17.75	2.1 $\times \;10^{-11}$	1.2 $\times \;10^{-11}$	2249
	1	19.09	2.1 $\times \;10^{-11}$	1.2 $\times \;10^{-11}$	2249
	1 $\times \;10^{-60}$	16.19	2.1 $\times \;10^{-11}$	1.2 $\times \;10^{-11}$	2249
GFLM-II	0.01	-	-	-	-
	0.001	4378.44	1.9 $\times \;10^{-14}$	2.8 $\times \;10^{-15}$	498,055
GFDN_BE	0.01	-	-	-	-
	0.001	64,524.93	2.0 $\times \;10^{-16}$	7.1 $\times \;10^{-15}$	498,637
GFDN_BF	0.01	-	-	-	-
	0.001	5241.62	4.7 $\times \;10^{-4}$	3.0 $\times \;10^{-4}$	500,000
DSFDN_LF	0.1	-	-	-	-
	0.01	448.04	6.0 $\times \;10^{-14}$	3.4 $\times \;10^{-14}$	53,146
DSFDN_SI	1	-	-	-	-
	0.1	48.66	2.1 $\times \;10^{-14}$	2.0 $\times \;10^{-16}$	5206
	0.01	476.76	1.2 $\times \;10^{-13}$	6.7 $\times \;10^{-14}$	53,148
DSFAL_SI	0.1	-	-	-	-
	0.01	1342.61	1.2 $\times \;10^{-9}$	1.3 $\times \;10^{-10}$	180,068
PCG	-	45.55	3.2 $\times \;10^{-13}$	5.4 $\times \;10^{-14}$	4159

**Table 13:** Numerical results with different strong interactions and fast rotations.

Ω **(**g** = 1000)**		**1**	**2**	**2.5**	**5**
ASNGF-I	CPU (s)	14,003	15,409	17,697	21,356
	#iter	1,865,136	2,052,409	2,357,160	2,844,523
	*E*	11.6586	−3.7805	−23.9484	−522.0957
ASNGF-II	CPU (s)	16,482	16,985	17,074	20,767
	#iter	2,195,327	2,262,324	2,274,179	2,766,070
	*E*	11.6586	−3.7805	−23.9484	−522.0957
**Ω (g = 10,000)**		**1.5**	**3**	**4**	**5**
ASNGF-I	CPU (s)	21,253	21,200	31,284	35,434
	#iter	2,830,804	2,823,744	4,166,888	677,979
	*E*	53.2634	−23.4771	−172.7135	−485.0218
ASNGF-II	CPU (s)	19,032	20,210	28,943	40,351
	#iter	2,534,975	2,691,880	3,855,076	5,374,569
	*E*	53.2634	−23.4771	−172.7135	−485.0218

## Data Availability

The original contributions presented in this study are included in the article. Further inquiries can be directed to the corresponding author.

## Appendix A

### Appendix A.1. MATLAB Code of the ASNGF Method for Example 1

Below is a sample MATLAB R2023a code of the ASNGF-I method, which computes the central vortex state in Example 1.

S = 2; g = −30; gamma = pi; tau = 1;
R = 20; N = 10∗R; alpha1 = tau^2; alpha2 = tau^2;
N1 = N+1; [x,w] = legslb(N1); r = R/2∗(x+1);
phi_n = 1/sqrt(pi∗factorial(S)).∗r.^S.∗exp(−r.^2/2);
% coefficient matrix A, B, C
A1 = 4∗(0:N−2)’+6; Am1 = 2∗(0:N−2)’+4; Ap1 = 2∗(−1:N−3)’+4;
A = spdiags([Am1,A1,Ap1],−1:1,N1−2,N1−2);
B1 = zeros(N−1,1); Bm1 = B1;
for i = 0:N−2
      B1(i+1) = 2∗(2∗i+3)/(i+1)/(i+2);
      Bm1(i+1) = −2/(i+2);
end
Bp1 = Bm1; Bp1(end) = []; Bp1 = [0;Bp1];
B = spdiags([Bm1,B1,Bp1],−1:1,N1−2,N1−2);
C1 = zeros(N−1,1); Cm1 = C1; Cm2 = C1; Cm3 = C1;
for i = 0:N−2
      C1(i+1) = 2/(2∗i+1)+2/(2∗i+5);
      Cm1(i+1) = 2/(2∗i+1)/(2∗i+5)+2∗(i+3)/(2∗i+5)/(2∗i+7);
      Cm2(i+1) = −2/(2∗i+5);
      Cm3(i+1) = −2∗(i+3)/(2∗i+5)/(2∗i+7);
end
Cp1 = Cm1; Cp1(end) = []; Cp1 = [0;Cp1];
Cp2 = Cm2; Cp2(end−1:end) = []; Cp2 = [0;0;Cp2];
Cp3 = Cm3; Cp3(end−2:end) = []; Cp3 = [0;0;0;Cp3];
C = spdiags([Cm3,Cm2,Cm1,C1,Cp1,Cp2,Cp3],−3:3,N1−2,N1−2);
% compute coefficient matrix AH for magnetic field H
a = zeros(N1−2,1); b = zeros(N1−2,1);
for k = 0:N−2
      a(k+1) = (2∗k+3)/(k+2)^2/(log(R)∗(k+1)∗(k+3)−1);
      b(k+1) = (k+1)^2/(k+2)^2∗(log(R)∗k∗(k+2)−1) ...
             /(1−log(R)∗(k+1)∗(k+3));
end
AH = zeros(N−1,1); AHm1 = AH;
for k = 0:N−2
      AH(k+1) = (2+2∗k)∗a(k+1)+(4∗k+6)∗b(k+1)+(2∗k+4)∗a(k+1) ...
               ∗b(k+1);
      AHm1(k+1) = (2∗k+4)∗b(k+1);
end
AHp1 = AHm1; AHp1(end) = []; AHp1 = [0;AHp1];
AH = spdiags([AHm1,AH,AHp1],−1:1,N1−2,N1−2);
% memory allocation
hatphi_n = zeros(N−1,1); hatphi_dotn = zeros(N−1,1);
legphi_1 = zeros(N1,1); legHhat = zeros(N1,1);
hatg = zeros(N−1,1); rhsH = zeros(N−1,1);
E = zeros(2∗1e+3,1); Err = zeros(2∗1e+3,1);
tol = 1e−10; time = 0; err = 1;
while err>tol
      time = time+1;
      legphi_n = ledisctran(N1,x,w,phi_n,0);
      hatphi_n(1) = legphi_n(1); hatphi_n(2) = legphi_n(2);
      for i = 3:N−1
            hatphi_n(i) = hatphi_n(i−2)+legphi_n(i);
      end
      % compute magnetic field H
      gamphi2 = gamma∗abs(phi_n).^2;
      leggamphi2 = ledisctran(N1,x,w,gamphi2,0);
      rhsH(1) = (1+a(1)/3)∗2∗leggamphi2(1)+(1+a(1)+2∗b(1)/5) ...
            ∗2/3∗leggamphi2(2)+(b(1)+2∗a(1)/3)∗2/5∗leggamphi2(3) ...
            +3∗b(1)/5∗2/7∗leggamphi2(4);
    for j = 1:N−3
            rhsH(j+1) = 2∗j/(4∗j^2−1)∗leggamphi2(j)+(1+a(j+1) ...
                          ∗(j+1)/(2∗j+3))∗2/(2∗j+1)∗leggamphi2(j+1) ...
                          +(a(j+1)+(j+1)/(2∗j+1) +b(j+1)∗(j+2)/(2∗j+5)) ...
                          ∗2/(2∗j+3)∗leggamphi2(j+2)+(b(j+1) +a(j+1) ...
                          ∗(j+2)/(2∗j+3))∗2/(2∗j+5)∗leggamphi2(j+3) ...
                          +b(j+1)∗(j+3)/(2∗j+5)∗2/(2∗j+7) ...
                          ∗leggamphi2(j+4);
    end
    rhsH(N−1) = 2∗(N−2)/(4∗(N−2)^2−1)∗leggamphi2(N−2) ...
            +(1+a(N−1)∗(N−1)/(2∗(N−2)+3))∗2/(2∗(N−2)+1) ...
            ∗leggamphi2(N−1) +(a(N−1)+(N−1)/(2∗(N−2)+1) ...
            +b(N−1)∗N/(2∗(N−2)+5))∗2/(2∗(N−2)+3)∗leggamphi2(N)...
            +(b(N−1)+a(N−1)∗N/(2∗(N−2)+3))∗2 ...
            /(2∗(N−2)+5)∗leggamphi2(N1);
    Hhat = −R^2/4∗(AH\rhsH);
    legHhat(1) = Hhat(1); legHhat(2) = Hhat(2)+a(1)∗Hhat(1);
    for i = 3:N−1
            legHhat(i) = Hhat(i)+a(i−1)∗Hhat(i−1)+b(i−2)∗Hhat(i−2);
    end
    legHhat(N) = a(N−1)∗Hhat(N−1)+b(N−2)∗Hhat(N−2);
    legHhat(N1) = b(N−1)∗Hhat(N−1);
    H = ledisctran(N1,x,w,legHhat,1);
    % nonlinear term
    Nl = −g∗abs(phi_n).^2+H; g1 = Nl.∗phi_n;
    legg = ledisctran(N1,x,w,g1,0);
    hatg(1) = legg(1); hatg(2) = legg(2);
    for i = 3:N−1
            hatg(i) = hatg(i−2)+legg(i);
    end
    kenitic = sum((pi∗(A+S^2∗B)∗hatphi_n).∗hatphi_n);
    E_int = sum((pi∗R^2/4∗C∗hatg).∗hatphi_n);
    E(time) = kenitic−E_int; mu_n = kenitic−2∗E_int;
    % residual
    hat_rn = C\(2/R^2∗(A+S^2∗B)∗hatphi_n−C∗(hatg+mu_n∗hatphi_n));
    err = sqrt(pi∗R^2/2∗sum(hat_rn.∗(C∗hat_rn)));
    Err(time) = err;
    if err<tol
            break
    end
    alpha = max(−0.5∗max(Nl+mu_n),0);
    alpha0 = 5/(time∗tau)∗alpha1;
    % coefficient matrix
    AS = (alpha0+alpha1/tau+alpha∗tau)∗C+2/R^2∗(2∗alpha2/tau ...
            +tau)∗(A+S^2∗B);
    hatphi_dot1 = AS\(2/R^2∗(A+S^2∗B)∗(−hatphi_n+2∗alpha2/tau ...
            ∗hatphi_dotn)+C∗(mu_n∗hatphi_n+hatg+alpha1/tau ...
            ∗hatphi_dotn));
    hatphi_1 = hatphi_n+tau∗hatphi_dot1;
    normphi_1 = sqrt(sum(pi∗R^2/2∗hatphi_1.∗(C∗hatphi_1)));
    legphi_1(1) = hatphi_1(1); legphi_1(2) = hatphi_1(2);
    for i = 3:N−1
            legphi_1(i) = hatphi_1(i)−hatphi_1(i−2);
    end
    legphi_1(N) = −hatphi_1(N−2); legphi_1(N1) = −hatphi_1(N−1);
    % normalization
    phi_1 = ledisctran(N1,x,w,legphi_1,1);
    phi_1 = phi_1/normphi_1;
    phi_n = phi_1; hatphi_dotn = hatphi_dot1;
end

### Appendix A.2. MATLAB Code of the ASNGF-I for Rapidly Rotating GPE

Below is a sample MATLAB R2023a code of the ASNGF-I method for the stationary state in Section 4.

h  =  1/2^4; R = 20; N  =  R/h;
x  =  −R/2:h:R/2−h; x = x’; y = x; [X,Y]  =  meshgrid(x,y);
Omega = 2; g  =  1000;
chi  =  1.2; kappa  =  0.3; gamma  =  1;
gammax  =  1; gammay  =  1;
V  =  (1−chi)∗(gammax^2∗X.^2+gammay^2∗Y.^2)+ kappa/4∗(X.^2 ...
            +Y.^2).^2;
phi_n = 1/sqrt(pi).∗exp(−(X.^2+Y.^2)/2);
mu  =  −N/2:N/2−1; mu  =  fftshift(mu)’;
mu  =  2∗pi∗mu/R; nv  =  mu;
[Mu,Nv]  =  meshgrid(mu,nv); LaplaceOperator  =  Mu.^2+Nv.^2;
f = zeros(N,N); hatphidotn = zeros(N,N);
tau = 1; alpha1 = 10∗tau^2; alpha2 = 0.1∗alpha1; coeff  =  5;
err = 1; time  =  1; tol = 1e−6;
while err>tol
    if Omega < 2 && time < 2000
            Omega  =  5;
    else
            Omega  =  1;
    end
    fftphin = fft2(phi_n); rho = abs(phi_n).^2;
    ft  =  1/2∗LaplaceOperator.∗fftphin;
    st  =  (V+g∗rho).∗phi_n + Omega∗(Y.∗ifft2(Mu.∗fftphin) ...
             −X.∗ifft2(Nv.∗fftphin));
    L = h^2∗sum(sum(1/N^2∗ft.∗conj(fftphin) + conj(phi_n).∗st));
    err  =  max(max(abs(ifft2(ft) + st − L∗phi_n)));
      if time > 5e+6
            break
      end
      alpha = 1/2∗max(max(max(abs(V+g∗rho+Omega∗pi∗N−L))),0);
      alpha0 = coeff∗alpha1/(time∗tau);
      fftphi1 = ((alpha0/tau+L+alpha).∗fftphin +1/tau∗(alpha1...
            +alpha2∗LaplaceOperator).∗(fftphin/tau+hatphidotn) ...
            −fft2(st))./(alpha0/tau+alpha1/tau^2+(alpha2/tau^2 ...
            +0.5)∗LaplaceOperator+alpha);
      hatphidot1 = (fftphi1−fftphin)/tau;
      phi_1 = ifft2(fftphi1);
      phi_n1 =  phi_1/h/norm(phi_1,’fro’);
      phi_n = phi_n1;
      hatphidotn  =  hatphidot1;
      time = time + 1;
end
